# Computational Analysis of Sequence Editability in the Theophylline RNA Aptamer as a Functional RNA Module

**DOI:** 10.3390/ijms27167228

**Published:** 2026-08-13

**Authors:** Aamir Aman, Leonhard Sidl, Nitchakan Darai, Peter Wolschann, Thanyada Rungrotmongkol, Michael T. Wolfinger

**Affiliations:** 1 Program in Bioinformatics and Computational Biology, College of Interdisciplinary and Integrative Studies, Chulalongkorn University, Bangkok 10330, Thailand; 2 Department of Theoretical Chemistry, University of Vienna, Währinger Straße 17, 1090 Vienna, Austria; leonhard.paul.sidl@univie.ac.at (L.S.);; 3 Research Group Bioinformatics and Computational Biology, University of Vienna, Währinger Straße 29, 1090 Vienna, Austria; 4 Vienna Doctoral School in Chemistry (DoSChem), University of Vienna, Währinger Straße 42, 1090 Vienna, Austria; 5 RNA Forecast e.U., 1140 Vienna, Austria; 6 Futuristic Science Research Center, School of Science, Walailak University, Nakhon Si Thammarat 80160, Thailand; 7 Research Center for Theoretical Simulation and Applied Research in Bioscience and Sensing, Walailak University, Thasala, Nakhon Si Thammarat 80160, Thailand; 8 Center of Excellence in Structural and Computational Biology, Department of Biochemistry, Faculty of Science, Chulalongkorn University, Bangkok 10330, Thailand

**Keywords:** theophylline, caffeine, RNA aptamer, modified RNA aptamer, MD simulation, waterswap

## Abstract

RNA aptamers are often used as ligand-recognition modules in engineered RNA systems, but integration into larger RNA constructs can influence stability and ligand binding. As a result, aptamer sequences may need to be adapted to new environments while preserving essential properties. Here, we examine this sequence editability problem for the theophylline RNA aptamer. Starting from the experimentally determined structure, we introduced targeted mutations in peripheral structural elements while leaving the recognition site unchanged. The native aptamer, mutated variants, a Mg^2+^-depleted system, and a caffeine-bound control were analyzed using three independent 1 μs molecular dynamics simulations. Binding energetics were estimated with multiple end-point as well as alchemical free energy approaches. Results were interpreted together with base pair stability, the conformational landscape of the binding pocket, and per-nucleotide energy contributions. This allows us to predict whether an edit is tolerated or disruptive. Some mutations retained structural and energetic profiles comparable to the native aptamer, whereas others reduced ligand affinity by propagating structural distortions into the binding pocket. These results show that sequence changes outside the binding site can modulate ligand binding indirectly, and that the ligand interaction network is useful for evaluating edited aptamers. The introduced workflow provides a novel combination of established computational strategies for efficient in silico screening of aptamer variants before experimental testing and can be integrated into the design of larger RNA structures. This works particularly well when an experimental structure is available and the tested mutations are small enough not to disrupt the folding pathway.

## 1. Introduction

RNA function is often linked to the formation of defined secondary and tertiary structures, as well as to conformational changes between alternative structural states. Such structural dynamics are central to many biological processes, including splicing, translation, and catalysis [1]. In addition, many RNAs interact directly with small organic molecules, which has made structured RNAs useful both as molecular targets and as components for sensing and regulatory applications [2]. Short ligand-binding RNAs, commonly referred to as aptamers, can be selected or engineered to recognize specific target molecules with high affinity and selectivity. Ligand binding can stabilize particular RNA conformations or shift conformational equilibria [3], allowing aptamers to be coupled to functional readout domains. This principle has been used in fluorescent aptamer-based sensors [3,4], as well as in engineered regulatory RNAs that modulate biological processes such as translation or gene expression [5,6]. The most common method for identifying aptamers against novel targets is SELEX [7,8,9], where large RNA libraries are iteratively enriched for molecules that bind the target ligand.

Aptamers are frequently used as modular ligand-recognition domains in synthetic RNA systems. In many applications, the aptamer is combined with an additional RNA element that provides a functional output, for example, a ribozyme, an expression platform, or a fluorescent reporter. This modular design strategy has been successful in many cases, but it also introduces a practical constraint. The aptamer is no longer isolated, but becomes part of a larger structural context which can influence folding and ligand binding. Thus, the sequence of an aptamer domain cannot always be transferred unchanged into the new context. In some designs, spacer elements are introduced to reduce interference between domains, whereas in others the aptamer sequence itself has to be adapted [10,11,12]. The relevant design problem is therefore not only whether an aptamer binds its ligand in the selected sequence, but also which sequence changes can be introduced without disrupting ligand recognition. In this regard, we define sequence editability as the degree of sequence flexibility possible without losing function, which for aptamers corresponds to binding affinity.

This issue is usually addressed either experimentally or by rational design. In empirical approaches, randomized sequence regions are screened or selected for retained function. In rational approaches, sequence changes are introduced based on secondary structure models, known structural constraints, or previous mutational data, followed by experimental validation [10]. Computational methods are useful in this setting because they can reduce the number of variants that need to be tested experimentally.

Several efficient algorithms are available to predict RNA secondary structures, base-pairing probabilities, and RNA–RNA interactions [13,14,15]. These methods are well suited for identifying unwanted base-pairing interactions or secondary structure changes based on the presence of a ligand. However, they provide only limited information on whether a sequence change preserves the three-dimensional ligand-binding architecture of an aptamer, and therefore its binding affinity. This is particularly important for small-molecule aptamers, where ligand recognition depends on local stacking interactions, hydrogen bonding patterns, ion stabilization, and the precise orientation of the ligand within the binding pocket.

The 33-nucleotide theophylline aptamer mTCT8-4 [16] serves as an effective model system to explore this challenge. It is one of the best characterized synthetic RNA aptamers and has been used extensively as a ligand-responsive component in engineered RNA systems [17], also due to its high selectivity for theophylline over structurally related xanthines, especially caffeine. High-resolution structural data are available for the theophylline-bound aptamer [18], and previous structural and biochemical studies have identified key features of the binding site, including ligand-specific hydrogen bonds, stacking interactions, metal-ion contributions, and structural motifs that mediate discrimination against caffeine [19,20]. The aptamer has also been incorporated into ligand-responsive ribozymes [21,22] and engineered riboswitches that control translation or gene expression [6,23,24].

Previous work has already established which residues and interactions are essential for ligand recognition in the native theophylline aptamer. However, far less is known about which changes in the surrounding scaffold, which makes up a large majority of the aptamer, are tolerated without loss of binding competence. This distinction is important for design applications. If the binding pocket is kept fixed, peripheral helices may provide a high degree of sequence flexibility that can be used to adapt the aptamer to new RNA contexts. While mutations in those regions are computationally more tractable, they can still affect ligand binding indirectly by changing helix stability, ion coordination, or the relative positioning of binding-pocket residues.

**Figure 1:**
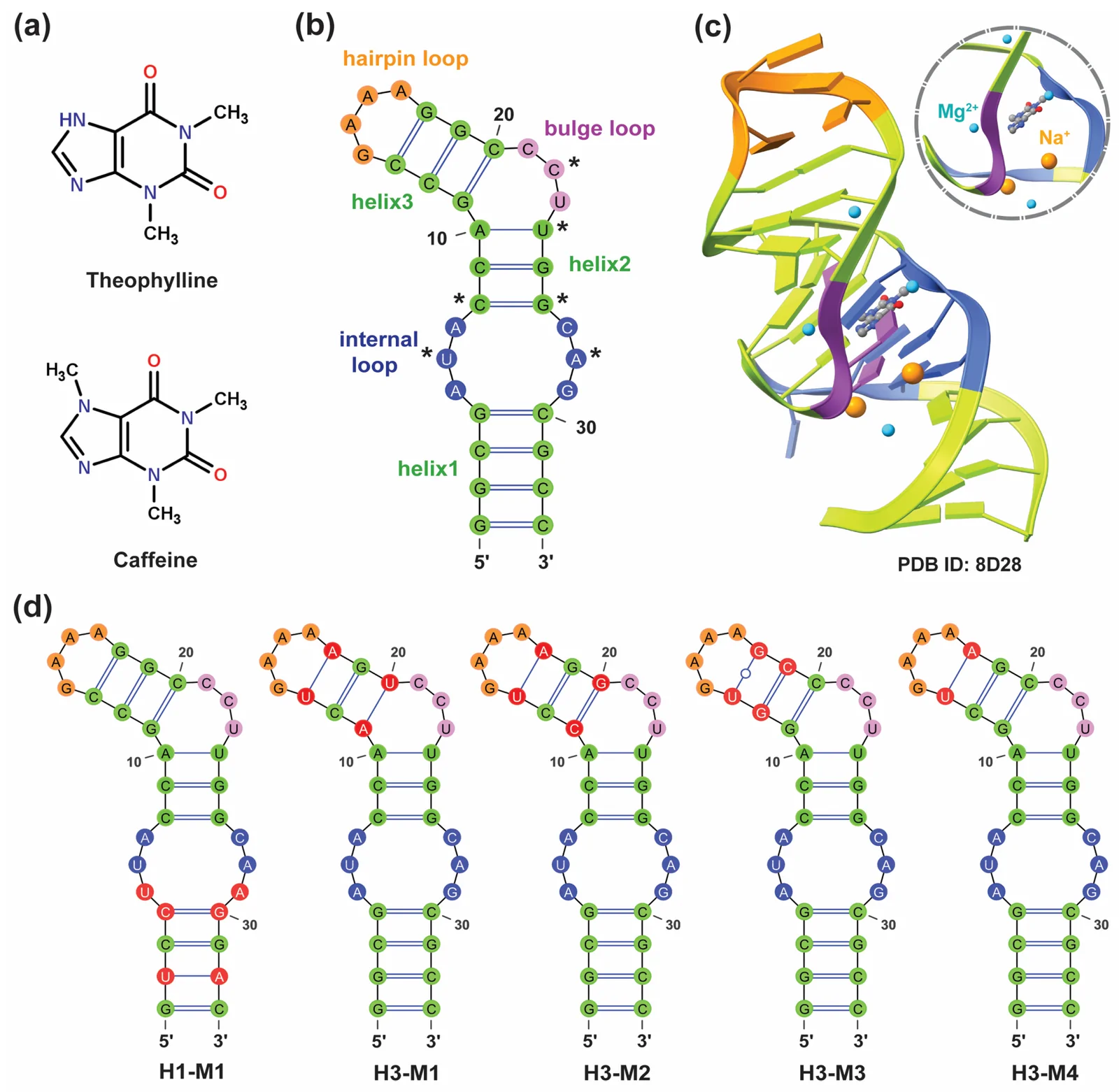
Overview of the theophylline aptamer variants analyzed in this study. (**a**) Chemical structures of theophylline and caffeine. (**b**) Secondary structure of the theophylline RNA aptamer, with the main structural elements indicated. Asterisks (*) denote the nucleotides that directly interact with the theophylline ligand. The binding site was kept unchanged in all designed variants. (**c**) Three-dimensional structure of the theophylline-bound aptamer (PDB ID: 8D28), showing the bound ligand and coordinating ions. The inset highlights the ligand-binding pocket. (**d**) Secondary structures of the modified aptamer variants. Mutated scaffold positions are shown in red, while the ligand-recognition site was preserved.

In this study, we showcase a novel combination of in silico methods to evaluate the peripheral sequence editability of the theophylline aptamer using molecular dynamics (MD) simulations and free energy calculations. We use the experimentally determined theophylline-bound structure as a reference and introduce targeted mutations in peripheral structural elements, while preserving the nucleotides with established ligand interactions (Figure 1). This approach permits a higher degree of sequence variability, compared to exclusively focusing on the binding domain. The selected variants were designed to probe two different types of scaffold modification: sequence changes in helix 3, which test the influence of regions adjacent to the binding pocket, and an extension of helix 1, which tests whether local compaction can be introduced without compromising ligand recognition. For each system, we performed three independent 1 μs all-atom MD simulations. We compared the native theophylline-bound aptamer, mutated aptamer variants, a Mg^2+^-depleted system, and a caffeine-bound control. Binding free energies were estimated using molecular mechanics–Generalized Born surface area (MM-GBSA), molecular mechanics–Poisson Boltzmann surface area (MM-PBSA), quantum mechanics/molecular mechanics–Generalized Born surface area (QM/MM-GBSA), and WaterSwap [25,26,27,28,29,30,31]. We further analyzed base pair probabilities, ligand contacts, per-nucleotide energy contributions, solvent accessibility, and binding pocket conformational dynamics.

The aim of this work is not to provide an exhaustive mutational map of the theophylline aptamer. Instead, we ask whether we can predict the classifications of a limited set of rationally chosen scaffold mutations based on their ability to preserve ligand recognition by comparing structural stability, relative binding energies, and the persistence of key ligand–pocket interactions. This provides a computational basis for evaluating edits to the theophylline aptamer and for screening candidates for experimental validation, and complements existing RNA design tools.

## 2. Results

### 2.1. Design of Peripheral Scaffold Edits

The mutations selected for further analysis were placed in structural elements adjacent to the theophylline binding pocket, while the nucleotides directly involved in ligand recognition were kept unchanged (Figure 1d). This design allowed us to probe sequence variation in the aptamer scaffold without deliberately altering the established recognition site. The introduced mutations were selected based on changes that are desirable when designing alternative interactions of the aptamer with neighboring regions such as readout or regulatory domains. The two helices of the native theophylline binding aptamer outside the binding site, helix 1 and helix 3, are composed exclusively of G-C pairs. This makes the design of competing interactions particularly challenging due to the extreme stability of G-C helices. Therefore, we selected mutants that introduce A-U and G-U base pairs, which generally also reduce the free energy of the native fold. Four variants, denoted H3-M1 to H3-M4, test sequence variability within helix 3. Variants H3-M2 and H3-M4 each introduce an A-U pair, while not drastically reducing the free energy of the native fold at the secondary structure level (−10.9 kcal mol^−1^ and −11.0 kcal mol^−1^ compared to −13.0 kcal mol^−1^). The H3-M1 variant introduces two A-U pairs and retains the native fold of the minimum free energy (MFE) structure with a noticeably lower free energy of −9.3 kcal mol^−1^. Finally, H3-M3 introduces a G-U wobble pair, which is particularly useful for designing more stable competing interactions by allowing the introduction of an alternative G-C pair. To probe sequence editability within helix 1, the H1-M1 variant adds one A-U pair and swaps one G-C pair. We furthermore replace the non-canonical interaction between positions 5 and 29, classified as cis Watson–Crick/Watson–Crick (cWW) in the Leontis–Westhof classification [32], with a canonical A-U pair, leading to an improvement in free energy.

Before analyzing the MD trajectories, we first evaluated whether the designed sequences were compatible with the native secondary structure. Base pairing probability dot plots show that the main base pairs of the native aptamer remain dominant in most variants (Figure S1). The main deviations were observed in the edited regions themselves. H1-M1 showed increased probability for an extended helix 1, consistent with the intended local compaction. For the helix 3 variants, only minor and local changes could be observed. Thus, the mutations did not globally disrupt the secondary structure, but introduced local distortions that are expected to affect the stability of the scaffold surrounding the binding site.

### 2.2. Peripheral Mutations Reveal Helix-Dependent Scaffold Stability

**Figure 2:**
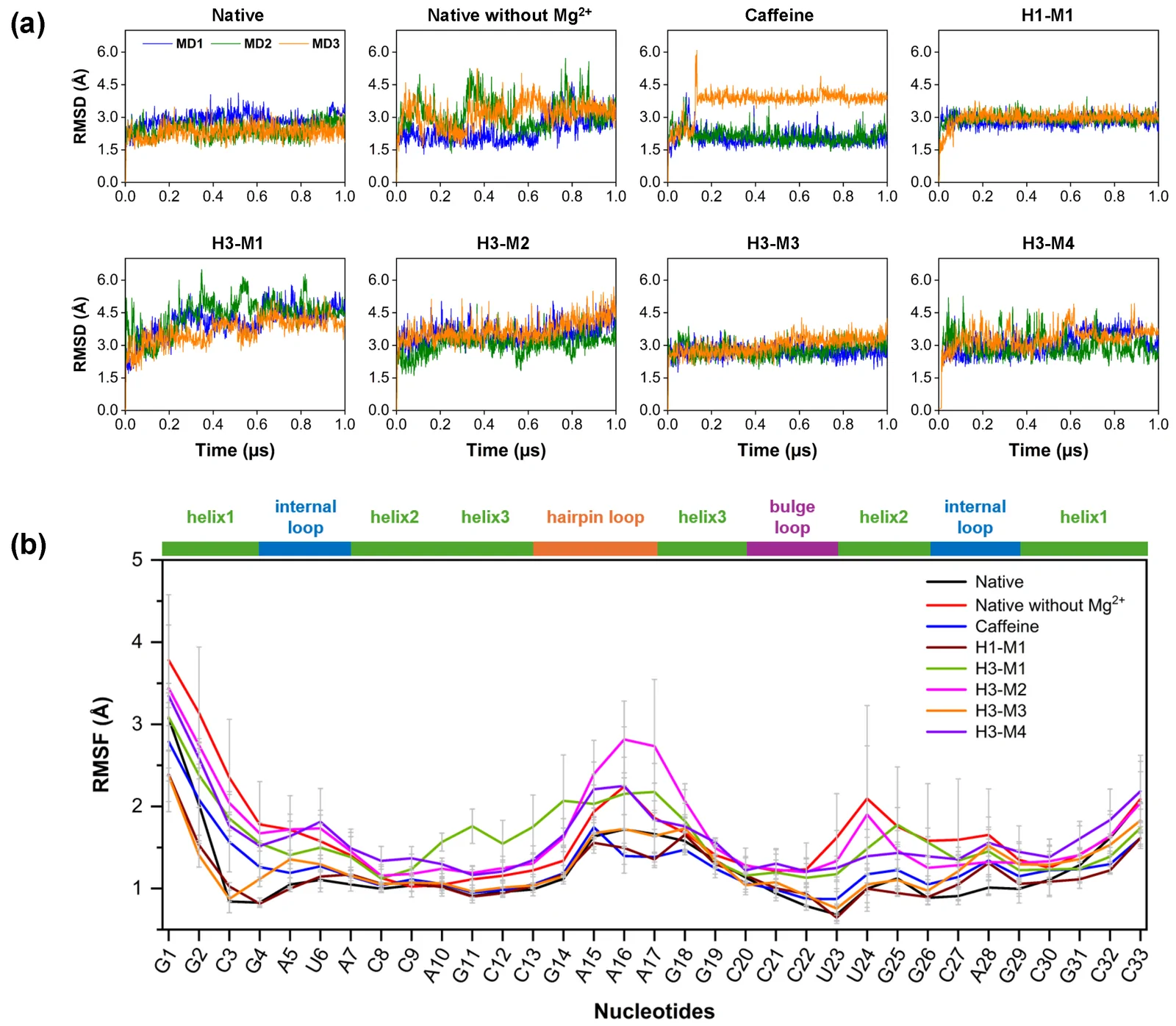
Conformational stability and local flexibility of native and modified aptamer complexes. (**a**) Backbone RMSD profiles from three independent 1 μs MD simulations per system. (**b**) Per-nucleotide RMSF values were calculated over the final 0.8–1.0 µs and averaged over the three replicates. The color bar above the plot indicates the corresponding secondary structure elements. Error bars indicate standard deviations across replicates. For consistency, nucleotide numbering on the x-axis corresponds to the native theophylline bound aptamer (PDB ID: 8D28) and is used as a reference for all variants.

The structural stability of the native aptamer and the modified complexes was first monitored by backbone RMSD over three independent 1 μs MD simulations (Figure 2a). The native theophylline-bound aptamer, including the crystallographic Mg^2+^ ions, equilibrated within the first 0.1–0.2 μs and remained stable during the remaining simulation time, with RMSD values mainly between 1.5 and 3.5 Å. Removal of Mg^2+^ ions led to slightly larger fluctuations and occasional deviations above 4 Å. This indicates a partial destabilization in the absence of divalent ions, although part of the effect could also be attributed to a more general ionic strength effect. The caffeine-bound system showed stronger fluctuations. In one replicate, caffeine left the binding pocket early in the simulation and remained associated with the terminal region of the aptamer until the end of the trajectory. Among the modified aptamers, H1-M1 and H3-M3 showed the lowest RMSD fluctuations and remained close to the behavior of the native theophylline-bound aptamer. H3-M1, H3-M2, and H3-M4 showed more variable trajectories. All systems reached stable conformational sampling by approximately 800 ns. For this reason, all subsequent structural and energetic analyses were performed on the last 0.8–1.0 μs of each trajectory.

The distance between U24:N3 and the ligand was used as an additional measure of ligand positioning in the binding pocket (Figure S2). In the native theophylline-bound aptamer, the U24:N3-theophylline contact remained persistent across all three replicates, with an average distance of approximately 3.0 Å and an occupancy above 99%. In contrast, the corresponding U24:N3-caffeine distance fluctuated between 5 and 10 Å for the two simulations where caffeine remained in the binding pocket, with only rare instances of approaches below the hydrogen bond cutoff. These trajectories are consistent with a rotated and less stable caffeine-binding mode. For the remaining trajectory, the distance increased to 20–25 Å as caffeine exited the binding pocket and associated with the terminal region of the stem.

Per-nucleotide RMSF values were used to identify regions in which the scaffold mutations affected local stability (Figure 2b). For all systems, terminal nucleotides showed the increased mobility expected for the closing regions of helices. The native aptamer and the more stable modified variants showed lower fluctuations than the Mg^2+^-depleted system and the more dynamic helix 3 variants. More pronounced differences were observed in the hairpin loop region, especially around G14–G19. H3-M2 showed the largest fluctuations in this region, followed by the Mg^2+^-depleted aptamer, H3-M4, and H3-M1. Additional increases were observed around helix 2 and the bulge-loop region U23-U24, most prominently in the Mg^2+^-depleted system and in H3-M2. H3-M1 also showed elevated fluctuations in helix 3 (G10–C13), suggesting that the mutations introduced into the helix reduced its stability, with the destabilizing effects propagating toward the adjacent hairpin region.

To assess whether the simulations reached equilibrium, we computed the cumulative mean RMSD as a function of simulation time for each replicate (Figure S3a). For most systems, the running mean converged within the first 0.2–0.3 μs and remained flat through the analyzed window, indicating that the last 0.8–1.0 μs are representative of the equilibrated ensemble. The three systems with the largest raw RMSD fluctuations, H3-M1, H3-M2, and H3-M4, showed correspondingly slower and less complete convergence, consistent with the increased structural variability already noted for these variants. Notably, the single caffeine replicate, in which the ligand dissociated from the binding pocket, is clearly distinguishable in this analysis as a running mean that fails to plateau, independently corroborating the ligand-escape event identified from visual inspection of the trajectory. This RMSD-based convergence assessment was complemented by comparing per-nucleotide RMSF profiles calculated separately for each replicate over the same 0.8–1.0 μs window (Figure S3b). The three replicates reproduced closely overlapping flexibility profiles for most systems, with the largest inter-replicate spread localized to the hairpin loop and helix 3 already identified as sites of increased local flexibility (Figure 2b).

Base pair hydrogen bond occupancies provided a complementary view of scaffold stability (Figure S4). The overall base pairing pattern was largely preserved, with local differences between helices. Helix 1 remained largely stable across all systems, although the introduced A-U pair in H1-M1 exerted a slight destabilizing effect. The strongest differences were observed in helix 3, where especially H3-M1 and H3-M4 showed reduced hydrogen bond occupancies at the base pairs mutated to A-U. These results align with the expected effects of the introduced mutations.

### 2.3. Free Energy Calculations Distinguish Tolerated and Disruptive Scaffold Edits

**Figure 3:**
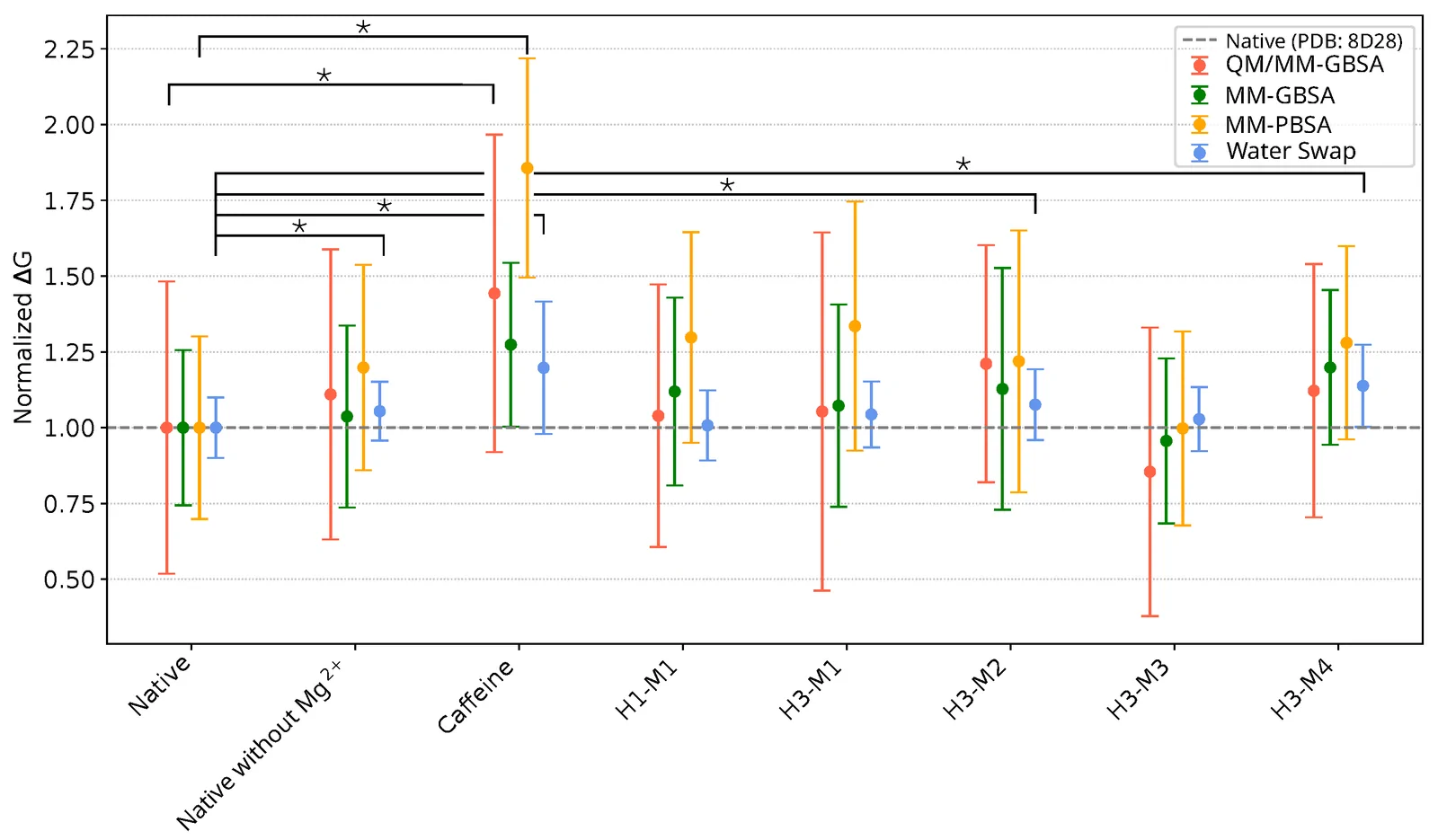
Relative binding energy trends across free energy methods. Binding free energies obtained from MM-GBSA, MM-PBSA, QM/MM-GBSA, and WaterSwap were normalized to the corresponding value of the native theophylline-bound aptamer to compare relative trends across methods with different absolute energy scales. The dashed horizontal line marks the native reference. Points represent mean values and error bars indicate standard deviations calculated from the three independent MD replicates. To facilitate comparison across methods with different absolute energy scales, energies on the y-axis were normalized relative to the native aptamer. A transformation of (1−y)+1 was then applied so that lower values indicate more favorable binding energies. Statistical significance compared to the native reference was computed using Welch’s *t*-test (p<0.05) and marked by asterisks.

Binding free energies were calculated from the equilibrated 0.8–1.0 µs portion of each trajectory using three endpoint approaches (MM-GBSA, MM-PBSA, and QM/MM-GBSA) together with the WaterSwap method. Although all four approaches estimate the binding free energy ΔG, including the entropy term, they employ substantially different approximations. The endpoint methods use implicit-solvent models and include solute entropic contributions estimated by normal-mode analysis, whereas WaterSwap evaluates ligand–water exchange through an alchemical procedure without an explicit solute entropy term. Consequently, the absolute ΔG values obtained from the different methods should not be compared directly. Instead, each result was evaluated relative to that of the native theophylline-bound aptamer, allowing trends that were reproduced across multiple computational approaches to be identified (Figure 3). The corresponding absolute energies are given in Table S1.

The three endpoint methods produced broadly consistent relative trends. The native aptamer and H3-M3 showed the most favorable energetic profiles, with overlapping uncertainty ranges across MM-GBSA, MM-PBSA, and QM/MM-GBSA. H3-M3 was therefore predicted to preserve a structural and energetic binding profile comparable to that of the native aptamer. H1-M1 and H3-M1 also showed binding free energies comparable to the native reference, with no consistent evidence of reduced binding across the endpoint methods. By contrast, H3-M2 and H3-M4 generally shifted toward less favorable binding energies, although the magnitude of the change varied among methods.

The caffeine-bound system showed significantly weaker binding than the native theophylline-bound aptamer across the endpoint calculations. In one replicate, caffeine dissociated from the binding pocket and remained associated with the terminal region of the RNA. Because endpoint binding free energy calculations assume a bound complex, this replicate was excluded from the energetic analysis. The remaining two replicates nevertheless produced substantially less favorable binding energies than the native theophylline complex, consistent with the altered binding orientation and reduced persistence of the native ligand-contact network. The Mg^2+^-depleted control also showed a modest shift toward less favorable binding, supporting the contribution of the crystallographic Mg^2+^ ions to stabilization of the ligand-bound architecture.

WaterSwap provided an independent assessment based on a substantially different computational framework. Given the systematic uncertainties inherent in binding free energy calculations and the sampling sensitivity of WaterSwap for flexible RNA–ligand complexes, we use the resulting ΔG values primarily as a semi-quantitative ranking of relative binding affinities rather than as precise estimates of absolute binding free energies. Although its absolute ΔG values were systematically more negative than those obtained from the endpoint methods, the relative trends were generally consistent. The caffeine-bound and Mg^2+^-depleted systems, as well as H3-M2 and H3-M4, showed significantly less favorable WaterSwap binding free energies than the native aptamer (*p* < 0.05), whereas no significant differences were detected for H1-M1, H3-M1, or H3-M3. Thus, the agreement across methods supports the predicted classification of H1-M1, H3-M1, and H3-M3 as tolerated scaffold edits and H3-M2 and H3-M4 as disruptive edits. More detailed results can be found in Figure S5.

Notably, H3-M1 exhibited pronounced loss of hydrogen-bond occupancy within the modified helix 3 but retained native-comparable overall and per-nucleotide binding energetics. This apparent discrepancy indicates that a local perturbation in the peripheral scaffold does not necessarily propagate into the ligand-binding pocket. The preservation of its principal ligand-facing contacts, ligand enclosure, and binding-pocket energetic profile therefore supports the classification of H3-M1 as a tolerated edit despite the structural changes within helix 3. Collectively, these results demonstrate that evaluation of aptamer editability should not rely on global scaffold stability alone but should integrate binding free energy trends with detailed analysis of the ligand-facing interaction network.

### 2.4. Tolerated Variants Preserve the Ligand-Recognition Interaction Network

**Figure 4:**
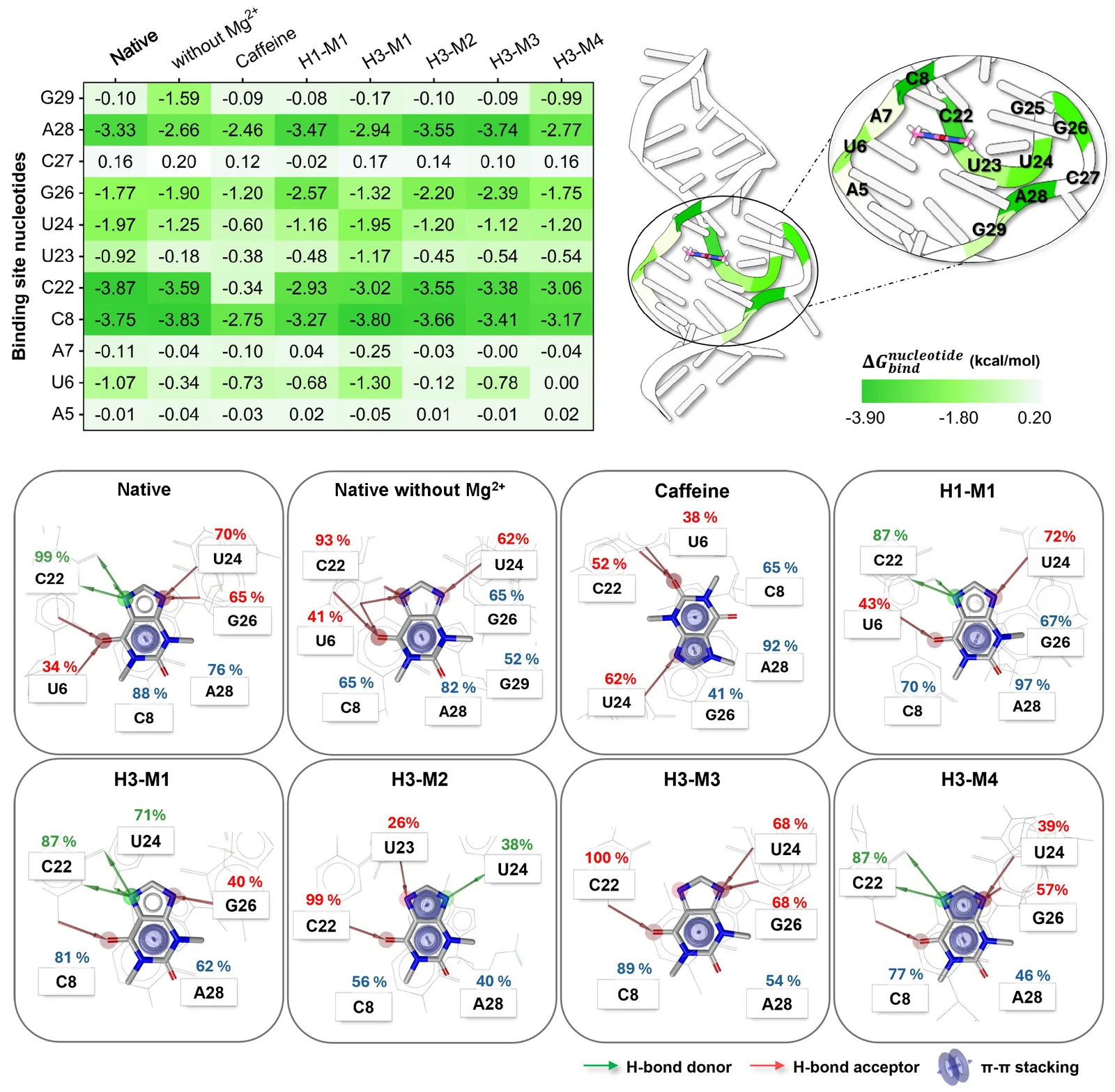
Ligand-facinginteraction network and per-nucleotide binding energy contributions. The upper left panel shows per-nucleotide MM-GBSA energy decomposition for nucleotides in and around the theophylline binding pocket, calculated over the last 0.8–1.0 μs of the three independent MD replicates. More negative values indicate more favorable ligand interactions. The structural inset highlights the analyzed binding pocket nucleotides. Nucleotide numbering follows the native aptamer sequence and is used as a reference for all variants. The upper right panel shows a close overview of the binding pocket nucleotides. The lower panels show ligand-interaction diagrams derived from the equilibrated trajectories. Green arrows indicate hydrogen bond donor interactions, red arrows indicate hydrogen bond acceptor interactions, and purple symbols indicate π-π stacking contacts. Percentages indicate the occupancy of each interaction during the analyzed trajectory window.

To identify how the scaffold mutations affected ligand recognition, we decomposed the MM-GBSA interaction energies by nucleotide for residues in and around the binding pocket (Figure 4). The analyzed residues include the bulge-loop nucleotides C22 and U23, the internal-loop residues A5, U6, A7, C27, A28, and G29, and the helical-interface residues C8, U24, and G26. In the native aptamer, C22, C8, and A28 made the strongest contributions to theophylline binding with values of −3.87, −3.75, and −3.33 kcal mol^−1^, respectively. U6, A7, U23, U24, G26, and G29 contributed more moderately. C27 showed little direct contribution, consistent with its limited contact with the ligand. The caffeine-bound system showed a strongly reduced interaction profile. The contribution of C22 decreased to −0.34 kcal mol^−1^ and that of U24 to −0.60 kcal mol^−1^, consistent with the loss of the native ligand-contact pattern. Among the scaffold variants, H1-M1, H3-M1, and H3-M3 preserved the native per-nucleotide profile, retaining strong contributions from C22 and stacking interactions with C8 and A28. Conversely, H3-M2 and H3-M4 showed reduced contributions around U23 and U24, although this was partly compensated by stronger A28 and G26 contributions. These patterns further support the classification into tolerated (H1-M1, H3-M1, H3-M3) and disruptive (H3-M2, H3-M4) mutants.

To characterize the specific non-covalent interactions formed between theophylline and key nucleotides within the aptamer binding pocket, LigandScout [33] 3D interaction diagrams were generated. In the native aptamer, theophylline formed a complete interaction pattern with high occupancy consisting of hydrogen bonding with C22 (99%), U24 (70%), G26 (65%), as well as π-π stacking with C8 (88%) and A28 (76%). The Mg^2+^-depleted system retained much of this pattern, but showed a lower U24 acceptor occupancy and an additional G29 stacking interaction. Caffeine adopted a rotated and less productive interaction pattern, with reduced C22, U24, and G26 contacts and a different balance of stacking interactions. H1-M1 and H3-M3 retained the native interaction network most closely. Both variants preserved the principal hydrogen-bonding and stacking contacts, explaining their near-native energetic behavior. H3-M1 retained the ligand contacts, albeit at reduced occupancy. Together with the significant disruptions in the hydrogen-bond patterns of helix 3, this further substantiates that peripheral structural changes are not always propagated into the binding pocket. Compared to that, H3-M2 showed a more substantial rearrangement, including a shift of the acceptor contact from U24 to U23 and reduced C8 and A28 stacking occupancies. H3-M4 also showed reduced U24 acceptor contacts and weaker interactions with G26 as well as with stacking partners. Thus, our simulations suggest that the variants with weaker binding signatures did not disrupt the ligand by mutating the binding site itself, but by introducing peripheral scaffold changes that altered the geometry and persistence of binding interactions.

### 2.5. Binding Pocket Conformational Landscape

**Figure 5:**
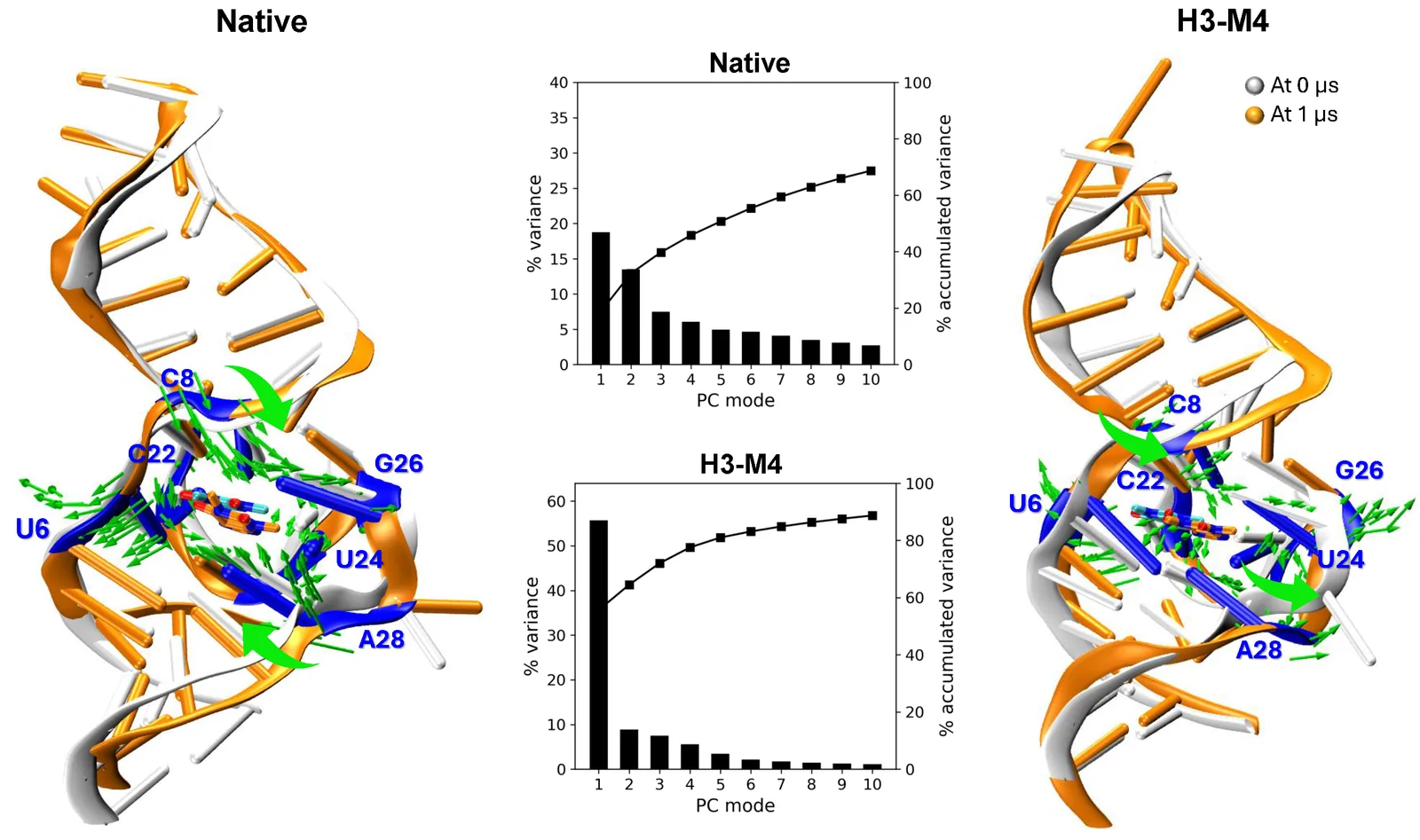
Principal component analysis (PCA) of binding pocket conformational dynamics for the native theophylline aptamer and the H3-M4 mutant. The left and right panels show the superimposed porcupine plot of the first (grey, at 0 μs) and last (orange, at 1 μs) simulation snapshots of the native theophylline-bound aptamer and H3-M4, respectively, with green arrows representing PC1 eigenvectors, where the arrowhead indicates the direction of movement, and the arrow length reflects the magnitude of the motion. Key binding pocket residues (U6, C8, C22, U24, G26, and A28) are labeled in blue. The theophylline ligand is shown in stick representation at the center of the binding pocket (0 μs and 1 μs snapshots are represented by cyan and orange colors, respectively). Middle panels display the scree plots for the native aptamer (top) and H3-M4 (bottom), showing the percentage of variance explained by each principal component (PC1–PC10) and the cumulative variance.

To characterize how scaffold changes influence the dominant modes of conformational motion within the theophylline recognition site, principal component analysis (PCA) was performed on the heavy atoms of the six conserved binding pocket nucleotides (U6, C8, C22, U24, G26, and A28) across all simulation replicates, using the last 0.8–1.0 µs of each trajectory. The PC1 eigenvectors were visualized as porcupine plots using VMD 2.0 [34,35], with the superimposed initial (grey, 0 μs) and final (orange, 1 μs) simulation snapshots presented in (Figure 5) for the native aptamer and the H3-M4 mutant. In both complexes, PC1 accounted for approximately 19 % of the total variance, with the remainder distributed broadly across subsequent modes. The porcupine plot revealed moderately sized inward-directed eigenvectors at C8, C22, U24, and A28 maintaining the spatial convergence of key recognition residues around the ligand. In contrast, PC1 of the H3-M4 mutant captured approximately 57% of the total variance, a threefold increase relative to the native aptamer, indicating that the binding pocket dynamics are dominated by a single directional motion. The large outward-directed eigenvectors concentrated at A28 and U24 are consistent with the reduced ligand contacts and weakened binding free energies documented for this mutant, providing a conformational basis for its classification as a disruptive edit.

Analysis of the remaining systems revealed a systematic relationship between PC1 dominance and binding pocket interaction integrity (Figure S6). The native complex in the absence of Mg^2+^ showed a PC1 contribution of approximately 50%, with inward-directed eigenvectors, indicating reduced but preserved pocket stability upon Mg^2+^ removal. In the caffeine-bound complex, PC1 contributed approximately 55% of total variance, and the porcupine plot reveals inward-directed eigenvectors with a clearly rotated orientation relative to the initial structure, reflecting the rotated binding mode adopted by caffeine within the recognition cavity. Among the helix 3 variants, H3-M1 showed a PC1 contribution of approximately 40%, with outward-directed eigenvectors of small magnitude at U24 and notable structural deviation at helix 3, suggesting partial destabilization of the scaffold adjacent to the binding pocket with only limited impact on the ligand-facing residues. H3-M2 displayed the highest PC1 contribution among all systems (∼65%), with eigenvectors concentrated at C8 and U24, consistent with the shifted acceptor contact from U24 to U23 and reduced stacking occupancies observed in the interaction analysis. In contrast, H1-M1 displayed the most distributed variance profile (∼27% for PC1), closely resembling the native aptamer and supporting its classification as a tolerated edit that preserves multi-modal binding pocket sampling. H3-M3 showed an intermediate PC1 contribution (∼30%) alongside notably large eigenvector magnitudes at multiple pocket residues, suggesting that this variant accesses a broad conformational ensemble while retaining the key ligand-facing interaction contacts.

The modified aptamers sampled broader and more displaced pocket ensembles than the native aptamer. The PCA analysis suggests that disruptive variants are associated with dominant motions that increase pocket heterogeneity and displace key binding residues away from the native interaction geometry and further supports the already established classification.

Ligand contacts with core nucleotides (C8, C22, U24, and A28 with a cutoff of 3.0 Å) and solvent-accessible surface area (SASA) were used to assess how well the ligand remained enclosed by the binding pocket (Figure S7). The native aptamer showed the highest average number of contacts (21.13±2.63) and the lowest ligand SASA (77.87±6.23 Å^2^), consistent with a compact and well-shielded binding pocket. Removal of Mg^2+^ slightly reduced the number of contacts (19.84±2.78) and increased the SASA (93.20±6.21 Å^2^). The caffeine-bound system showed fewer contacts (17.67±3.12) and a much higher SASA (143.44±7.50 Å^2^), reflecting poorer enclosure of the ligand. Among the theophylline-bound mutants, H3-M4 showed fewer contacts (19.80±4.02) and increased SASA (104.81±9.81 Å^2^), while the remaining variants were closer to the native aptamer. These descriptors are consistent with the free energy, interaction analyses, and PCA, with H1-M1 and H3-M3 retaining near-native binding signatures and H3-M1 showing more fluctuations in the SASA while not losing ligand contacts.

### 2.6. Integrated Classification of Aptamer Scaffold Edits

The combined structural, energetic, and interaction analyses support a predicted classification of the tested variants according to their compatibility with ligand recognition (Table 1). H1-M1 and H3-M3 retained near-native scaffold structures and binding signatures, preserved the main ligand-facing contacts, and are therefore predicted as tolerated edits.

For the H3-M1 variant, hydrogen bond analysis showed a significant distortion of helix 3. However, this did not propagate into the binding pocket, since absolute and per-nucleotide binding energy as well as the ligand-facing interaction network were comparable to the native aptamer. Furthermore, the binding pocket atom contacts were stable during simulations, and only small-magnitude outward-directed eigenvectors were observed. We therefore predict that the variant is tolerated despite structural deviations of the scaffold. Meanwhile, H3-M2 and H3-M4 show weaker binding signatures and perturbations of the ligand-facing interaction network, which can be classified as disruptive edits. The Mg^2+^-depleted and caffeine-bound systems served as controls for structural destabilization and ligand-specific disruption, respectively, resulting in lower binding affinities.

## 3. Discussion

In this study, we investigated how selected sequence modifications outside the conserved binding pocket affect the structural stability and ligand recognition properties of the theophylline RNA aptamer. The mutations were chosen in peripheral structural elements, while the nucleotides directly involved in theophylline recognition were kept unchanged. This setup allowed us to examine whether the surrounding scaffold of the aptamer can be modified without disrupting the molecular interactions that define the ligand-bound state. The theophylline aptamer is well suited for this question, since its binding pocket, metal ion stabilization, and discrimination against caffeine have previously been extensively characterized [16,18,19,20]. Our results therefore address a complementary aspect of aptamer design, namely the extent to which non-core nucleotides, making up a majority of the structure, can be altered while preserving the native recognition site.

Our approach extends the design toolkit for larger RNA constructs, such as RNA switches, where an aptamer structure is typically coupled with a regulatory domain. Existing strategies either preserve the full aptamer sequence, thereby limiting the design of alternative conformations in ligand-bound and ligand-free states [36], or constrain only the binding pocket, neglecting the influence of mutations in the surrounding scaffold on ligand recognition [37]. In contrast, the strategy presented here enables greater sequence flexibility while providing an in silico assessment of whether individual peripheral mutations are likely to disrupt aptamer function, thus allowing efficient prescreening of variants prior to experimental validation.

The use of classical MD simulations facilitates the application of this framework to a broad range of ligand-binding systems with available experimental structures. In practice, this prerequisite is readily satisfied for many commonly used aptamers, as experimentally determined tertiary structures are available for systems such as the theophylline, neomycin, and glutamine aptamers [17,38,39]. Restricting the analysis to experimentally determined RNA–ligand complexes avoids the considerable uncertainty associated with predicting ligand-bound RNA structures de novo. At the same time, the approach assumes that peripheral mutations do not substantially alter the folding landscape, either by changing the folding pathway in the ligand-bound state or by reducing the population of binding-competent conformations in its absence. Consequently, the method is best suited for variants containing a limited number of mutations and is not intended for designs expected to induce major structural rearrangements.

The employed free energy approaches yielded different binding energies, as expected from the underlying approximations of MM-GBSA, MM-PBSA, QM/MM-GBSA, and WaterSwap. However, the relative trends were consistent across the methods. The native aptamer, H1-M1, and H3-M3 formed the most favorable group of theophylline-bound systems, whereas H3-M2 and H3-M4 showed reduced binding signatures and broader energy distributions. H3-M1 showed a structural and energetic profile of the binding site comparable to the native aptamer despite changes in the interaction network within helix 3. These results indicate that the aptamer scaffold is not uniformly tolerant to sequence change and that a combination of analytic approaches is necessary for proper investigations. The classification of these variants based on the computational analyses should be interpreted as an in silico prioritization, suitable for prescreening potential candidates, rather than as a definitive affinity ranking.

The interaction analyses provide the molecular basis for these trends. In the native aptamer, theophylline is stabilized by a defined network of hydrogen bonding and stacking interactions, with C22, C8, and A28 contributing most strongly to the binding energy and additional contacts involving U24, G26 and U6. H1-M1 preserved this interaction pattern despite the modifications in helix 1, and H3-M1 as well as H3-M3 retained the main C8 and A28 stacking contributions with only minor changes in the surrounding contacts. By contrast, H3-M2 and H3-M4 showed clear perturbations of the ligand-facing interaction network. In H3-M2, the acceptor interaction shifted from U24 to U23 and stacking occupancies were reduced, while H3-M4 showed weakened contacts involving C22, C8, and U24. Thus, the disruptive variants did not directly modify the binding pocket, but affected ligand binding indirectly through changes in the scaffold that altered the geometry and stability of the binding pocket.

The directionality of PC1 eigenvectors provides a mechanistic interpretation of how scaffold mutations alter the theophylline recognition geometry. In the native aptamer, inward-directed eigenvectors at C8, C22, U24, and A28 describe a concerted pocket-closing motion that geometrically converges key recognition residues around the ligand, consistent with the persistent hydrogen bonding and π-π stacking occupancies observed for these nucleotides. The reversal of this directionality in H3-M4, where large outward-directed eigenvectors dominate at A28 and U24, indicates that helix 3 mutations shift the dominant conformational tendency of the pocket from convergent to divergent, directly disrupting the geometric arrangement required for a stable ligand coordination. An outward displacement of smaller magnitude was observed in H3-M1, indicating changes in the scaffold that did not fully propagate into the binding pocket. H3-M2 showed laterally displaced eigenvectors at C8 and U24, structurally consistent with the shifted acceptor contact from U24 to U23 documented in the interaction analysis. In contrast, H1-M1 and H3-M3 retained inward-directed eigenvectors closely resembling the native aptamer, providing a conformational basis for their classification as tolerated edits. These patterns establish that the distinction between tolerated and disruptive scaffold edits is reflected not only in conformational amplitude but in the geometric direction of dominant pocket motion, tolerated variants preserve inward convergence toward the ligand, while disruptive variants redirect this motion outward, destabilizing the recognition geometry without altering the global fold.

The comparison between the structural and energetic descriptors further suggests that preservation of the global fold alone is not sufficient to judge whether a sequence variant will retain ligand recognition. In H3-M2, reduced hydrogen bond occupancies in helix 3 propagated to the binding pocket, shifting ligand interactions from U24 to U23 and reducing stacking occupancies. Conversely, the destabilizing mutations of H3-M1, as indicated by hydrogen bond occupancy, did not result in a clear reduction in stability since the key C8/C22/A28 contacts were preserved. For the H3-M2 mutant, some shifts in the binding conformation seem to be tolerated. In this case, reduced interactions with U24 were likely compensated by stronger interactions with G26. This indicates that a modified aptamer does not necessarily need to reproduce the native conformational ensemble exactly, provided that the ligand-facing interaction pattern is maintained. When perturbations do propagate, they lead to reduced ligand enclosure and higher ligand SASA, indicating a more solvent-exposed binding site. This is best seen for H3-M4 (Figure S7) and is consistent with the outward directed pocket motions identified by PCA. For this reason, RMSD, RMSF, or secondary structure preservation alone are not sufficient criteria for evaluating aptamer variants, but need to be interpreted together with the persistence of the binding-pocket interaction network and free energy approximations.

The Mg^2+^-depleted and caffeine-bound systems served as useful controls for this interpretation. Removal of Mg^2+^ increased conformational variability and weakened parts of the interaction pattern around the binding pocket. Because Mg^2+^ removal alters both the specific divalent-coordination sites and the overall electrostatic screening of the system, this destabilization should be interpreted as a combined effect rather than an isolated measure of specific Mg^2+^-site contributions. This is consistent with earlier work identifying Mg^2+^ as important for the structural integrity of the theophylline-binding core [20]. Caffeine binding showed a different type of perturbation. Caffeine contains an additional methyl group at the N7 position relative to theophylline, which introduces steric clashes and alters hydrogen-bonding capacity within the binding pocket. Consequently, we observed that caffeine adopted a rotated binding pose compared to theophylline, lost several of the native stabilizing contacts, and in one replicate completely escaped from the binding pocket. This behavior is consistent with previous structural and NMR studies showing the extreme selectivity of the theophylline aptamer [19,20,40].

In the present study, we analyzed a selected set of rationally chosen mutations using a novel combination of in silico methods. Hence, the results do not represent a complete mutational map of the theophylline aptamer. In addition, all variants were modeled from the experimentally determined ligand-bound structure, and the simulations primarily probe the stability of the bound architecture rather than the full folding pathway or the initial ligand-binding event. The calculated binding energies should consequently be interpreted as relative indicators. Within these limits, the agreement between conformational stability, free energy trends, energy decomposition, ligand contacts, and solvent accessibility supports a consistent picture of scaffold-dependent sequence editability. The results suggest that peripheral sequence changes can be useful for adapting the aptamer to new structural contexts, but can modulate ligand recognition indirectly. Preservation of the ligand-facing interaction network is a good estimator of maintained binding competence and is therefore a useful criterion for prioritizing aptamer variants before experimental testing.

## 4. Materials and Methods

### 4.1. System Preparation

The crystal structure of the theophylline RNA aptamer in complex with theophylline, four Mg^2+^ ions, and two sodium ions was retrieved from the RCSB Protein Data Bank (PDB ID: 8D28) [41]. Crystallographic Mg^2+^ ions were retained in all systems except for the ion-depleted control. For the ion-depleted control, the four crystallographic Mg^2+^ ions were removed and the system was re-neutralized with additional Na^+^ counter-ions (30 Na^+^ in total). The overall ionic strength and counter-ion valency were therefore not matched to the Mg^2+^-containing system. The three-dimensional structure of caffeine was obtained from the PubChem database [42]. The protonation states of both theophylline and caffeine were examined using MarvinSketch 24.3.0 [43]. Under physiological conditions (pH 7.4), both compounds exist predominantly in their neutral forms; therefore, neutral protonation states were used throughout all simulations. Partial atomic charges for theophylline and caffeine were derived using the RESP (Restrained Electrostatic Potential) fitting procedure at the HF/6-31G* level using Gaussian16 [44], consistent with the General AMBER Force Field 2 (GAFF2) parameterization protocol [45]. Molecular docking using AutoDock Vina 1.2.0 [46] was performed to generate the complex between caffeine and the theophylline RNA aptamer. Mutations were introduced into the aptamer sequence by changing the crystal structure using the Web 3DNA 2.0 webserver [47] followed by 5000 steps of energy optimizations using QRNAS 0.3 [48].

### 4.2. MD Simulations

The RNA aptamer was described using AMBER RNA.OL3 force field [49], which provides improved RNA backbone and glycosidic (χ) torsional parameters compared with earlier ff99-based force fields, resulting in more accurate and stable RNA conformational sampling during molecular dynamics (MD) simulations. Theophylline and caffeine were parameterized using custom force field parameters, all prepared using the tLEaP module of AMBER22 [50]. The solute was neutralized by adding 22 Na^+^ counter-ions, described by the Joung–Cheatham ion parameters [51] and positioned on a Coulombic-potential grid to offset the net negative charge of the RNA backbone. The systems were then solvated in a rectangular TIP3P water box with a minimum 12 Å buffer between the solute and the box edge, adding 7628 water molecules. The retained Mg^2+^ ions were described using the Li–Merz 12-6 Lennard-Jones non-bonded parameters for divalent ions [52]. The added water was minimized using 1000 steps of the steepest descent method and 3000 steps of the conjugate gradient, with the solute (RNA, ligand, and ions) restrained by a harmonic force constant of 500 kcal·mol^−1^·Å^−2^ to relax the solvent. Subsequently, the entire system was minimized using the same procedure. All simulations assumed periodic boundary conditions and a 2 fs time step. Initially, the complexes were heated from 10 K to 310 K for 100 ps, followed by three individual all-atom MD simulations in the NPT ensemble at 310 K and 1 atm pressure, utilizing AMBER22. The SHAKE algorithm [53] was applied to constrain bonds involving hydrogen, and long-range electrostatic interactions were treated using the Particle Mesh Ewald (PME) [54] method with a cutoff distance of 12 Å. Three replicates of all-atom MD simulations were conducted for each system, with a duration of 1 μs.

### 4.3. Structural Stability Assessment and Binding Free Energy Calculations

To assess the structural stability of the simulated systems, backbone root mean square deviation (RMSD), cumulative mean RMSD and per-nucleotide root mean square fluctuation (RMSF) were calculated using the CPPTRAJ module [55] of AMBER22. Ligand–pocket intermolecular contacts and ligand solvent-accessible surface area (SASA) were calculated between all heavy atoms of the ligand and the binding pocket nucleotides using a distance cutoff of 3.0 Å, with SASA computed using the LCPO algorithm [56]. Hydrogen bond percentage occupancies were calculated for Watson–Crick base pairs within each helix using a donor–acceptor distance cutoff of 3.0 Å and a donor-hydrogen-acceptor angle cutoff of 135° [55]. Binding affinity was assessed through MM-GBSA, MM-PBSA, and QM/MM-GBSA-based binding free energy calculations using the MMPBSA.py 14.0 [57,58] module of AMBER22. For the caffeine-bound system, one replicate in which caffeine left the binding pocket was excluded from binding free energy calculations. For MM-GBSA, MM-PBSA, and QM/MM-GBSA calculations, 200 snapshots were extracted from the last 0.8–1.0 μs of each replicate. The GB model 2 (igb = 2) was employed for MM-GBSA calculations, and the dielectric constant was set to 1.0 for the solute interior. Per-nucleotide MM-GBSA energy decomposition was performed using the pairwise decomposition scheme (idecomp = 1) for the whole aptamer [57]. For QM/MM-GBSA calculations, the ligand and the key interacting nucleotide C22 were defined as the QM region and treated using the PM6 semiempirical method, while the remaining system was described at the molecular mechanics (MM) level. Entropy contributions for MM/GB(PB)SA, and QM/MM-GBSA were estimated using normal mode analysis (nmode) as implemented in MMPBSA.py 14.0 [25] leading to approximations of the binding free energy from all employed methods.

(1) ΔG=ΔH−TΔS

Statistical significance of binding free energy differences between each variant and the native theophylline bound aptamer was assessed using Welch’s *t*-test (p<0.05).

### 4.4. WaterSwap

Absolute binding free energies ΔG of theophylline bound to the native and mutated RNA aptamers, and of caffeine bound to the native RNA aptamer, were calculated using the WaterSwap method as implemented in the OpenBioSim/Sire molecular simulation framework [30]. To account for conformational sampling, 10 representative snapshots were extracted at equal intervals from the last 0.8–1.0 μs of each MD trajectory, again excluding one replica where caffeine left the binding pocket, using CPPTRAJ 6.29.13 [55], yielding one snapshot every 20 ns. Each snapshot was used as the starting configuration for an independent WaterSwap calculation, with the AMBER topology providing force field parameters for the solvated RNA–ligand complex. WaterSwap computes the absolute binding free energy by simultaneously decoupling the ligand from the binding site while coupling an equivalent cluster of water molecules in its place, along a λ-pathway ranging from λ=0 (ligand fully bound) to λ=1 (binding site fully occupied by water) [30]. Monte Carlo sampling was performed at each window via a Replica Exchange Thermodynamic Integration (RETI) scheme with Δλ=0.001 [59]. A flexibility cutoff of 15.0 Å around the ligand center was applied to define the mobile region of the system. Each calculation comprised 50,000 Monte Carlo equilibration moves followed by 1000 production iterations at 298.15 K. Binding free energies were estimated at each snapshot using three independent estimators: Bennett Acceptance Ratio (BAR) [60], free energy perturbation (FEP) [61], and thermodynamic integration (TI). The final reported binding free energy per replica was taken as the mean value ± standard deviation of all three estimators across all ten snapshots. To quantitatively assess whether each modified aptamer differs significantly from the native aptamer, paired two-sample *t*-tests were performed comparing the ΔG distributions of each variant against the native complex.

### 4.5. Principal Component Analysis

To capture the binding pocket conformational dynamics, principal component analysis (PCA) was performed on the Cartesian coordinates of the heavy atoms of six conserved binding pocket nucleotides (U6, C8, C22, U24, G26, and A28) across all simulation replicates using the last 0.8–1.0 μs of each trajectory. These six nucleotides directly line the theophylline recognition site and are structurally conserved across all native and modified aptamer sequences. Prior to analysis, all frames were superimposed onto a common reference structure to remove overall translational and rotational motion. The mass-weighted covariance matrix of atomic positional fluctuations was constructed and subsequently diagonalized to yield a set of orthogonal eigenvectors (principal components, PCs) and their associated eigenvalues. Each eigenvalue represents the mean-square displacement of the system projected along the corresponding PC, and was expressed as a fraction of the total variance to quantify its relative contribution to the overall conformational space sampled. The first ten PC modes were retained for analysis. The fractional variance explained by each individual mode, together with the cumulative variance as a function of mode index, were computed to assess the dimensionality of the dominant motions and to confirm that the leading PCs captured a sufficient proportion of the total conformational variance. All PCA calculations were performed using CPPTRAJ, and variance decomposition data were extracted from the eigenvalue fraction output file (evalfrac.dat). Variance scree plots were generated using Matplotlib 3.10.0 [62], displaying both the per-mode fractional variance and the cumulative variance for the first ten PC modes.

## Figures and Tables

**Table 1:** Summary of predicted scaffold editability classes inferred from the combined structural, energetic, and ligand-interaction analyses.

System Name	System Type	Main Observations	Interpretation
Native	Native reference (PDB ID: 8D28)	Stable fold, favorable binding energies, compact pocket, and complete C22/C8/A28-centered interaction network.	Reference
Native without Mg^2+^	Ion-depleted control	Increased structural variability, reduced pocket stability, and reduced binding strength.	Structural control
Caffeine	Ligand control	Rotated ligand pose, reduced native contacts, high SASA, and weakest binding.	Specificity control
H1-M1	Helix-1 scaffold edit	Stable RMSD, compact scaffold, near-native WaterSwap energy, and preserved ligand-facing contacts.	Tolerated edit
H3-M1	Helix-3 scaffold edit	Increased local flexibility within helix 1, but no loss of binding stability across all descriptors.	Tolerated edit
H3-M2	Helix-3 scaffold edit	Reduced helix/pocket stability, shifted and reduced ligand contacts, and weaker WaterSwap profile.	Disruptive edit
H3-M3	Helix-3 scaffold edit	Low RMSD fluctuations, binding energetics comparable to the native structure, and preserved C8/A28 stacking contributions.	Tolerated edit
H3-M4	Helix-3 scaffold edit	Weaker WaterSwap profile, reduced U24/G26/A28 contacts, shifted ligand interactions, and increased SASA.	Disruptive edit

## Data Availability

Input structures, parameter files, MD simulation protocol, analysis scripts, and simulation movies are available online: https://github.com/leonhards98/Theophylline-Aptamer_Editability (accessed on 7 August 2026). Full trajectories are available from the corresponding authors upon reasonable request.

## Supplementary Materials

The following supporting information can be downloaded at: https://www.mdpi.com/article/10.3390/ijms27167228/s1.

## Abbreviations

The following abbreviations are used in this manuscript:

MD	Molecular Dynamics
SD	Standard Deviation
RMSD	Root-Mean-Squared Deviation
RMSF	Root-Mean-Squared Fluctuation
SASA	Solvent-Accessible Surface Area
MM-GBSA	Molecular Mechanics/Generalized Born Surface Area
MM-PBSA	Molecular Mechanics/Poisson–Boltzmann Surface Area
QM/MM-GBSA	Quantum Mechanics/Molecular Mechanics-Generalized Born Surface Area
TI	Thermodynamic Integration
FEP	Free Energy Perturbation
BAR	Bennett’s Acceptance Ratio
PCA	Principal Component Analysis
MFE	Minimum Free Energy

## References

[1] Russell R., Zhuang X., Babcock H.P., Millett I.S., Doniach S., Chu S., Herschlag D. (2002). Exploring the folding landscape of a structured RNA. Proc. Natl. Acad. Sci. USA.

[2] Ilgu M., Nilsen-Hamilton M. (2016). Aptamers in analytics. Analyst.

[3] Cho E.J., Lee J.W., Ellington A.D. (2009). Applications of aptamers as sensors. Ann. Rev. Anal. Chem..

[4] Jhaveri S.D., Kirby R., Conrad R., Maglott E.J., Bowser M., Kennedy R.T., Glick G., Ellington A.D. (2000). Designed signaling aptamers that transduce molecular recognition to changes in fluorescence intensity. J. Am. Chem. Soc..

[5] Topp S., Gallivan J.P. (2010). Emerging applications of riboswitches in chemical biology. ACS Chem. Biol..

[6] Wittmann A., Suess B. (2012). Engineered riboswitches: Expanding researchers’ toolbox with synthetic RNA regulators. FEBS Lett..

[7] Ellington A.D., Szostak J.W. (1990). In vitro selection of RNA molecules that bind specific ligands. Nature.

[8] Tuerk C., Gold L. (1990). Systematic evolution of ligands by exponential enrichment: RNA ligands to bacteriophage T4 DNA polymerase. Science.

[9] Robertson D.L., Joyce G.F. (1990). Selection in vitro of an RNA enzyme that specifically cleaves single-stranded DNA. Nature.

[10] Soukup G.A., Breaker R.R. (1999). Design of allosteric hammerhead ribozymes activated by ligand-induced structure stabilization. Structure.

[11] Topp S., Reynoso C.M., Seeliger J.C., Goldlust I.S., Desai S.K., Murat D., Shen A., Puri A.W., Komeili A., Bertozzi C.R. (2010). Synthetic riboswitches that induce gene expression in diverse bacterial species. Appl. Environ. Microbiol..

[12] Desai S.K., Gallivan J.P. (2004). Genetic screens and selections for small molecules based on a synthetic riboswitch that activates protein translation. J. Am. Chem. Soc..

[13] Alkan F., Wenzel A., Palasca O., Kerpedjiev P., Rudebeck A.F., Stadler P.F., Hofacker I.L., Gorodkin J. (2017). RIsearch2: Suffix array-based large-scale prediction of RNA–RNA interactions and siRNA off-targets. Nucleic Acids Res..

[14] Lorenz R., Bernhart S.H., Höner zu Siederdissen C., Tafer H., Flamm C., Stadler P.F., Hofacker I.L. (2011). ViennaRNA Package 2.0. Algorithms Mol. Biol..

[15] Bernhart S.H., Tafer H., Mückstein U., Flamm C., Stadler P.F., Hofacker I.L. (2006). Partition function and base pairing probabilities of RNA heterodimers. Algorithms Mol. Biol..

[16] Jenison R.D., Gill S.C., Pardi A., Polisky B. (1994). High-resolution molecular discrimination by RNA. Science.

[17] Wrist A., Sun W., Summers R. (2020). The theophylline aptamer: 25 years as an important tool in cellular engineering research. ACS Synth. Biol..

[18] Menichelli E., Lam B.J., Wang Y., Wang V.S., Shaffer J., Tjhung K.F., Bursulaya B., Nguyen T.N., Vo T., Alper P.B. (2022). Discovery of small molecules that target a tertiary-structured RNA. Proc. Natl. Acad. Sci. USA.

[19] Zimmermann G.R., Jenison R.D., Wick C.L., Simorre J.P., Pardi A. (1997). Interlocking structural motifs mediate molecular discrimination by a theophylline-binding RNA. Nat. Struct. Biol..

[20] Zimmermann G.R., Wick C.L., ShieldsS T.P., Jenison R.D., Pardi A. (2000). Molecular interactions and metal binding in the theophylline-binding core of an RNA aptamer. RNA.

[21] Tang J., Breaker R.R. (1997). Rational design of allosteric ribozymes. Chem. Biol..

[22] Robertson M.P., Ellington A.D. (2000). Design and optimization of effector-activated ribozyme ligases. Nucleic Acids Res..

[23] Harvey I., Garneau P., Pelletier J. (2002). Inhibition of translation by RNA–small molecule interactions. RNA.

[24] Bayer T.S., Smolke C.D. (2005). Programmable ligand-controlled riboregulators of eukaryotic gene expression. Nat. Biotechnol..

[25] Miller B.R., McGee T.D., Swails J.M., Homeyer N., Gohlke H., Roitberg A.E. (2012). MMPBSA.py: An efficient program for end-state free energy calculations. J. Chem. Theory Comput..

[26] Li C., Du H., Zhang C., Huang W., Zhang X., Wang T., Jiang D., Hou T., Wang E. (2025). Comprehensive evaluation of End-Point free energy methods in DNA–Ligand interaction predictions. J. Chem. Inf. Model..

[27] Gouda H., Kuntz I.D., Case D.A., Kollman P.A. (2003). Free energy calculations for theophylline binding to an RNA aptamer: Comparison of MM-PBSA and thermodynamic integration methods. Biopolymers.

[28] Wang C., Greene D., Xiao L., Qi R., Luo R. (2018). Recent developments and applications of the MMPBSA method. Front. Mol. Biosci..

[29] Wang E., Sun H., Wang J., Wang Z., Liu H., Zhang J.Z., Hou T. (2019). End-point binding free energy calculation with MM/PBSA and MM/GBSA: Strategies and applications in drug design. Chem. Rev..

[30] Woods C.J., Malaisree M., Hannongbua S., Mulholland A.J. (2011). A water-swap reaction coordinate for the calculation of absolute protein–ligand binding free energies. J. Chem. Phys..

[31] Nutho B., Pengthaisong S., Tankrathok A., Lee V.S., Ketudat Cairns J.R., Rungrotmongkol T., Hannongbua S. (2020). Structural basis of specific glucoimidazole and mannoimidazole binding by Os3BGlu7. Biomolecules.

[32] Leontis N.B., Westhof E. (2001). Geometric nomenclature and classification of RNA base pairs. RNA.

[33] Wolber G., Langer T. (2005). LigandScout: 3-D pharmacophores derived from protein-bound ligands and their use as virtual screening filters. J. Chem. Inf. Model..

[34] Humphrey W., Dalke A., Schulten K. (1996). VMD–Visual Molecular Dynamics. J. Mol. Graph..

[35] Theoretical and Computational Biophysics Group (2024). VMD 2.0: Visual Molecular Dynamics.

[36] Wu M.J., Andreasson J.O., Kladwang W., Greenleaf W., Das R. (2019). Automated design of diverse stand-alone riboswitches. ACS Synth. Biol..

[37] Findeiß S., Hammer S., Wolfinger M.T., Kühnl F., Flamm C., Hofacker I.L. (2018). In silico design of ligand triggered RNA switches. Methods.

[38] Heel S.V., Juen F., Bartosik K., Micura R., Kreutz C., Breuker K. (2024). Resolving the intricate binding of neomycin B to multiple binding motifs of a neomycin-sensing riboswitch aptamer by native top-down mass spectrometry and NMR spectroscopy. Nucleic Acids Res..

[39] Ren A., Xue Y., Peselis A., Serganov A., Al-Hashimi H.M., Patel D.J. (2015). Structural and dynamic basis for low-affinity, high-selectivity binding of L-glutamine by the glutamine riboswitch. Cell Rep..

[40] Akhter S., Tang Z., Wang J., Haboro M., Holmstrom E.D., Wang J., Miao Y. (2024). Mechanism of ligand binding to theophylline RNA aptamer. J. Chem. Inf. Model..

[41] Berman H.M., Westbrook J., Feng Z., Gilliland G., Bhat T.N., Weissig H., Shindyalov I.N., Bourne P.E. (2000). The protein data bank. Nucleic Acids Res..

[42] Kim S., Chen J., Cheng T., Gindulyte A., He J., He S., Li Q., Shoemaker B., Thiessen P., Yu B. (2023). PubChem 2023 update. Nucleic Acids Res..

[43] ChemAxon (2024). MarvinSketch.

[44] Frisch M.E., Trucks G., Schlegel H.B., Scuseria G., Robb M., Cheeseman J., Scalmani G., Barone V., Petersson G., Nakatsuji H. (2016). Gaussian 16.

[45] Moriarty N.W., Case D.A., Liebschner D., Adams P.D. (2026). Validated ligand geometries for macromolecular refinement restraints and molecular-mechanics force fields. Acta Crystallogr. Sect. D Struct. Biol..

[46] Eberhardt J., Santos-Martins D., Tillack A., Forli S. (2021). AutoDock Vina 1.2.0: New Docking Methods, Expanded Force Field, and Python Bindings. J. Chem. Inf. Model..

[47] Li S., Olson W.K., Lu X.J. (2019). Web 3DNA 2.0 for the analysis, visualization, and modeling of 3D nucleic acid structures. Nucleic Acids Res..

[48] Stasiewicz J., Mukherjee S., Nithin C., Bujnicki J.M. (2019). QRNAS: Software tool for refinement of nucleic acid structures. BMC Struct. Biol..

[49] Zgarbová M., Otyepka M., Sponer J., Mladek A., Banas P., Cheatham T.E., Jurecka P. (2011). Refinement of the Cornell et al. nucleic acids force field based on reference quantum chemical calculations of glycosidic torsion profiles. J. Chem. Theory Comput..

[50] Case D., Aktulga H., Belfon K., Ben-Shalom I., Berryman J., Brozell S., Cerutti D., Cheatham T., Cisneros G., Cruzeiro V. (2022). Amber 2022.

[51] Joung I., Cheatham T. (2008). Determination of Alkali and Halide Monovalent Ion Parameters for Use in Explicitly Solvated Biomolecular Simulations. J. Phys. Chem. B.

[52] Li P., Merz K. (2013). Taking into Account the Ion-Induced Dipole Interaction in the Nonbonded Model of Ions. J. Chem. Theory Comput..

[53] Ryckaert J.P., Ciccotti G., Berendsen H.J. (1977). Numerical integration of the cartesian equations of motion of a system with constraints: Molecular dynamics of n-alkanes. J. Comput. Phys..

[54] Luty B.A., van Gunsteren W.F. (1996). Calculating electrostatic interactions using the particle–particle particle–mesh method with nonperiodic long-range interactions. J. Phys. Chem..

[55] Roe D., Cheatham T. (2013). PTRAJ and CPPTRAJ: Software for Processing and Analysis of Molecular Dynamics Trajectory Data. J. Chem. Theory Comput..

[56] Weiser J., Shenkin P., Still W. (1999). Approximate atomic surfaces from linear combinations of pairwise overlaps (LCPO). J. Comput. Chem..

[57] Genheden S., Ryde U. (2015). The MM/PBSA and MM/GBSA methods to estimate ligand-binding affinities. Expert Opin. Drug Discov..

[58] Phanich J., Threeracheep S., Kungwan N., Rungrotmongkol T., Hannongbua S. (2019). Glycan binding and specificity of viral influenza neuraminidases by classical molecular dynamics and replica exchange molecular dynamics simulations. J. Biomol. Struct. Dyn..

[59] Woods C.J., Malaisree M., Michel J., Long B., McIntosh-Smith S., Mulholland A.J. (2014). Rapid decomposition and visualisation of protein–ligand binding free energies by residue and by water. Faraday Discuss..

[60] Bennett C. (1976). Efficient estimation of free energy differences from Monte Carlo data. J. Comput. Phys..

[61] Zwanzig R. (1954). High-temperature equation of state by a perturbation method. I. Nonpolar gases. J. Chem. Phys..

[62] Hunter J. (2007). Matplotlib: A 2D graphics environment. Comput. Sci. Eng..

