# Supplementary material for: Computational Analysis of Sequence Editability in the Theophylline RNA Aptamer as a Functional RNA Module
Source: Int J Mol Sci. 2026 Aug 13;27(16):7228. doi: 10.3390/ijms27167228 (PMC13513662; doi:10.3390/ijms27167228)
Supplement: Supplementary file 1 [file ijms-27-07228-s001.zip › ijms-4409706-supplementary.pdf]

Computational Analysis of Sequence Editability in the Theophylline RNA Aptamer as a Functional RNA Module
# - Supplementary Data -

Aamir Aman[1,*], Leonhard Sidl[2,3,4,5,*], Nitchakan Darai[6,7], Peter Wolschann[2], Thanyada Rungrotmongkol[1, 8, †], and Michael T. Wolfinger[2,5, †]

[1]Program in Bioinformatics and Computational Biology, College of Interdisciplinary and Integrative Studies, Chulalongkorn University, Bangkok 10330, Thailand
[2]Department of Theoretical Chemistry, University of Vienna, Währinger Straße 17, 1090 Vienna, Austria
[3]Research Group Bioinformatics and Computational Biology, Faculty of Computer Science, University of Vienna, Währinger Straße 29, 1090 Vienna, Austria
[4]Vienna Doctoral School in Chemistry, University of Vienna, Währinger Straße 42, 1090 Vienna, Austria
[5]RNA Forecast e.U., Vienna, Austria
[6]Futuristic Science Research Center, School of Science, Walailak University, Nakhon Si Thammarat 80160, Thailand
[7]Research Center for Theoretical Simulation and Applied Research in Bioscience and Sensing, Walailak University, Thasala, Nakhon Si Thammarat 80160, Thailand
[8]Center of Excellence in Structural and Computational Biology, Department of Biochemistry, Faculty of Science, Chulalongkorn University, Bangkok 10330, Thailand

[*]A.A. and L.S. contributed equally to this work
[†]To whom correspondence should be addressed: thanyada.r@chula.ac. and michael.wolfinger@univie.ac.at

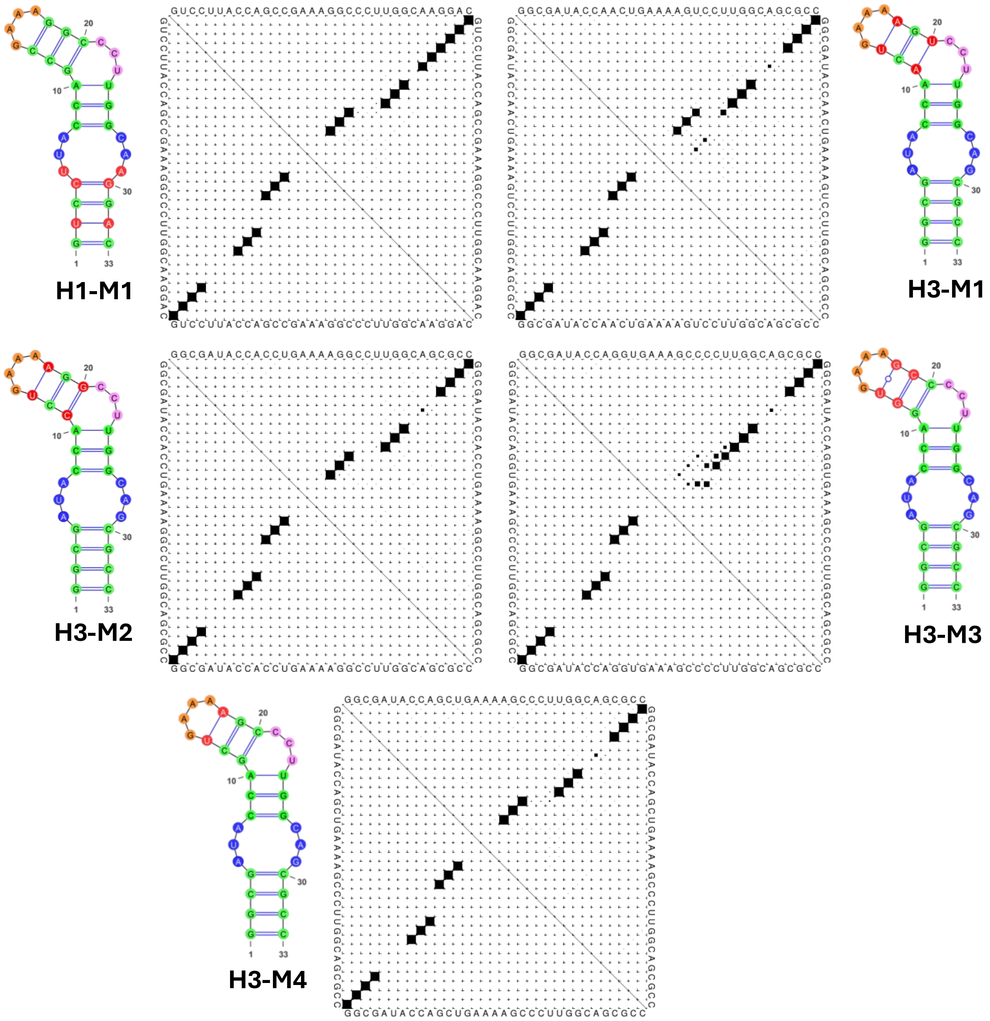

Figure S1: Dot-Plots of all mutated aptamers. The upper triangular section depicts the base pairing probabilities, with the area of the back square at position (i, j) corresponding to the probability of base i and j forming a base pair. The lower triangular section displays the reference structure of the unmodified RNA aptamer.

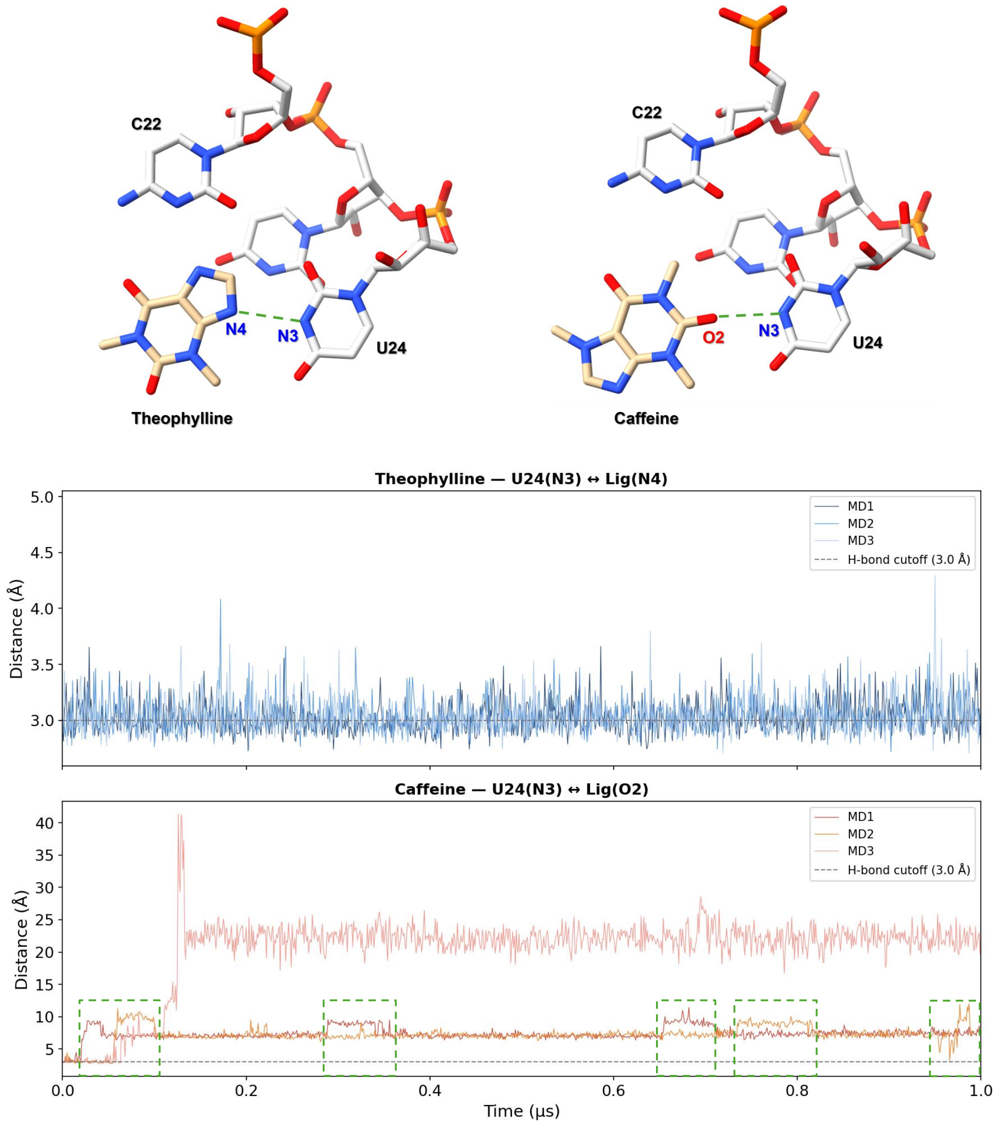

Figure S2: Key contact distance between U24:N3 and the ligands over a 1 µs MD simulation. Time evolution of the distance between the N3 atom of U24 and the key ligand atom for theophylline (N4, top) and caffeine (O2, bottom) across three independent MD replicates. The movement of caffeine inside a binding pocket is highlighted in green lines. The dashed gray line indicates the hydrogen bond distance cutoff (3.0 Å).

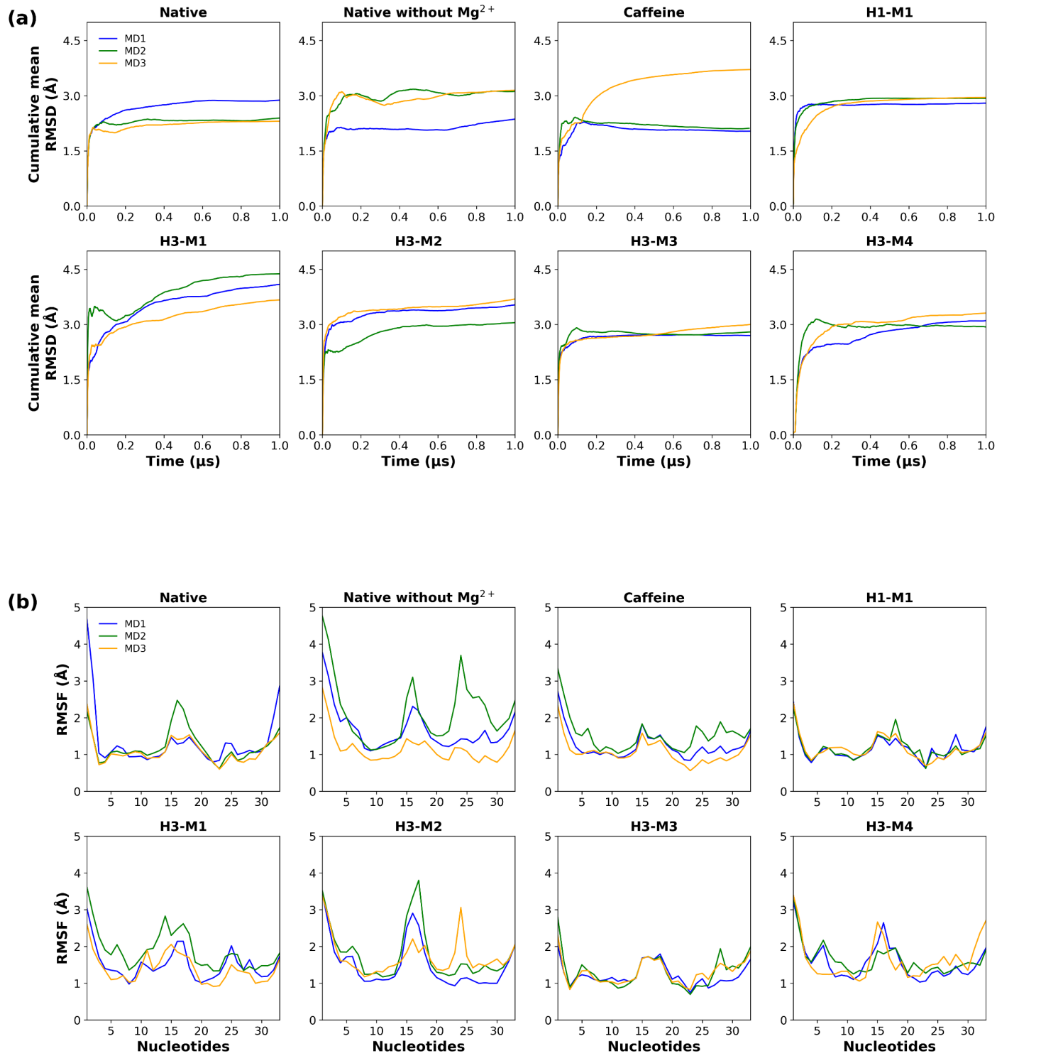

Figure S3: RMSD convergence and per-replicate RMSF comparison across independent simulations. (a) Cumulative mean backbone RMSD as a function of simulation time for each of the three independent 1 µs MD replicates (MD1, blue; MD2, green; MD3, orange) of all eight systems. (b) Per-nucleotide RMSF calculated separately for each of the three replicates over the last 0.8 - 1.0 µs of each trajectory.

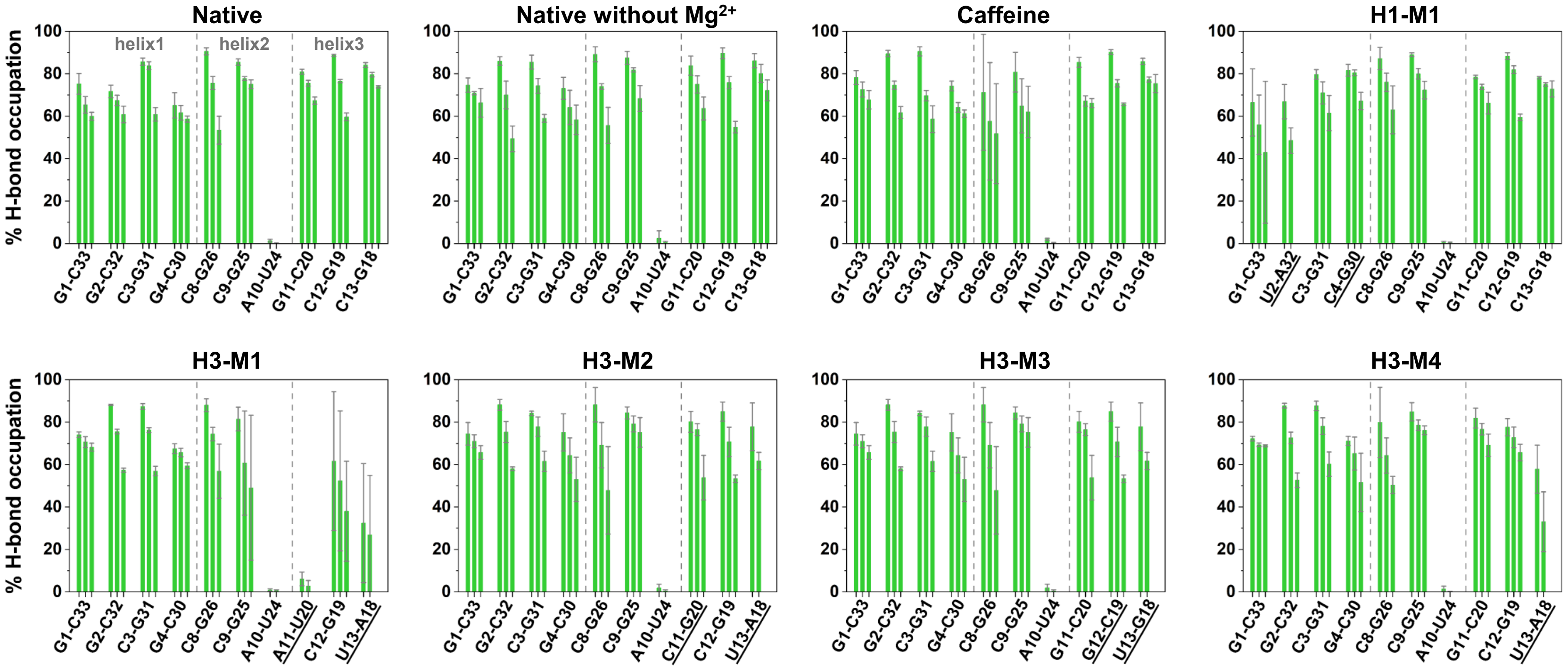

Figure S4: Hydrogen bond occupancies of helical base pairs in native and modified aptamer complexes. Occupancies were calculated over 0.8 - 1.0 μs of each trajectory and averaged over three independent MD replicates. Bars indicate the mean percentage of simulation time in which the corresponding hydrogen bond contact satisfied the geometric cutoff; error bars indicate standard deviations across replicates. Dashed vertical lines separate helix1, helix2, and helix3. Underlined labels indicate base pairs containing mutated nucleotides.

Table S1: Binding free energies including entropy (ΔG_{bind}, kcal mol^{-1}) of native and engineered RNA aptamer complexes calculated using MM-GBSA, MM-PBSA, QM/MM-GBSA, and WaterSwap methods from 0.8 - 1.0 µs of each simulation. Values are reported as mean ± SD from three independent simulations ($n = 3$). For the caffeine-bound system, the replicate in which the ligand dissociated from the binding pocket was excluded from the binding free energy calculations.

	**Classical MD**			**WaterSwap**
**Complex**	$\Delta G^{\text{MM-GBSA}}$	$\Delta G^{\text{MM-PBSA}}$	$\Delta G^{\text{QM/MM-GBSA}}$	ΔG
**Native (PDB ID: 8D28)**	-12.53 ± 4.15	-12.30 ± 4.19	-7.82 ± 4.05	-24.82 ± 2.47
**Native without Mg^{2+}**	-12.07 ± 4.01	-9.86 ± 4.30	-6.96 ± 3.81	-23.47 ± 2.41
**Caffeine**	-9.11 ± 3.88	-1.77 ± 5.30	-4.35 ± 4.23	-19.92 ± 5.42
**H1-M1**	-11.04 ± 4.58	-8.64 ± 4.79	-7.51 ± 4.36	-24.63 ± 2.88
**H3-M1**	-11.63 ± 4.69	-8.18 ± 5.85	-7.40 ± 4.93	-23.73 ± 2.70
**H3-M2**	-10.93 ± 5.29	-9.61 ± 6.40	-6.17 ± 3.32	-22.93 ± 2.90
**H3-M3**	-13.08 ± 3.86	-12.33 ± 4.57	-8.96 ± 4.16	-24.12 ± 2.62
**H3-M4**	-10.04 ± 4.10	-8.86 ± 5.49	-6.87 ± 3.90	-21.39 ± 3.36

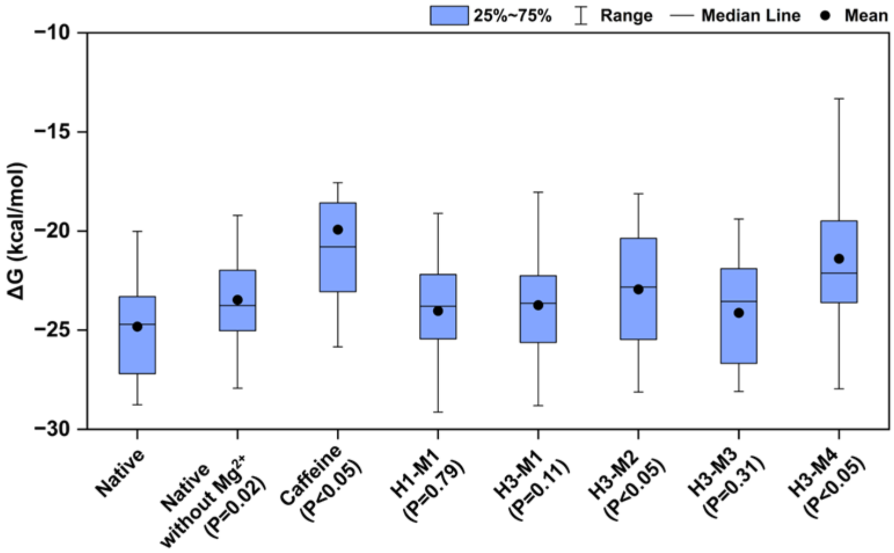

Figure S5: Distribution of WaterSwap binding free energies (ΔG, kcal mol^{-1}) for the native and mutant aptamers. All WaterSwap calculations were performed using TI, FEP, and BAR estimators applied to representative structures extracted from 0.8 - 1.0 µs of the triplicates. Box plots represent the interquartile range (25th–75th percentile), whiskers indicate the full range of values across the ten 20 ns trajectory segments, the horizontal line denotes the median, and filled black circles correspond to mean values. P-values below each system label indicate statistical significance of the difference relative to the native theophylline-aptamer complex.

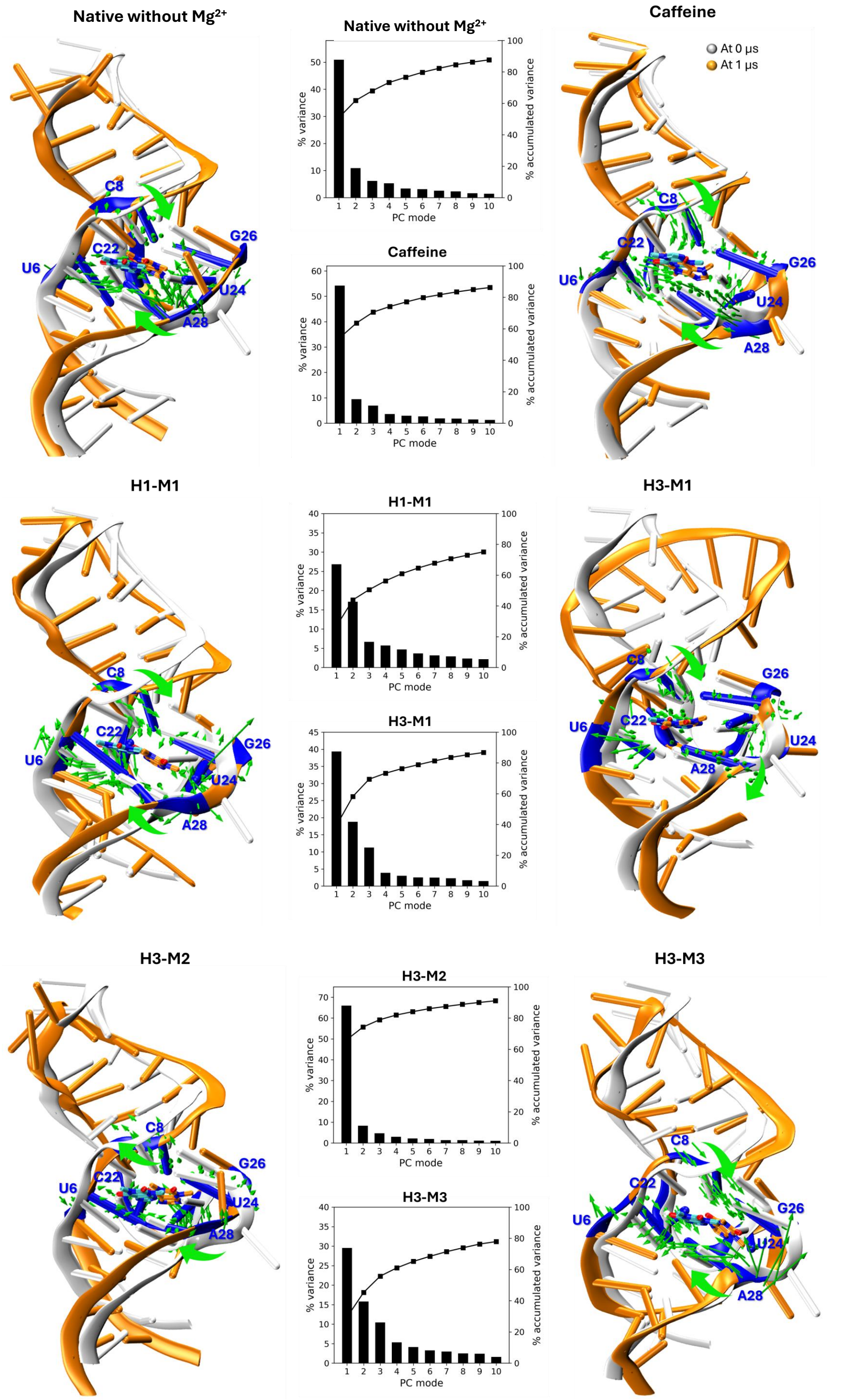

Figure S6: Principal component analysis (PCA) of binding pocket conformational dynamics across six RNA aptamer–ligand complexes. Each row displays the superimposed porcupine plot of initial (grey, at 0 μs) and final (orange, at 1 μs) simulation snapshots alongside the corresponding PC1 scree plots for two systems per row. Green arrows represent PC1 eigenvectors depicting the dominant collective motions of the six conserved binding pocket nucleotides (U6, C8, C22, U24, G26, and A28, labeled in blue), where the arrowhead indicates the direction of movement, and the arrow length reflects the magnitude of the motion. The ligand is shown in stick representation at the center of the binding pocket (0 μs and 1 μs snapshots are represented by cyan and orange colors, respectively). The middle column presents scree plots showing the percentage of variance explained by each principal component (PC1–PC10) and the cumulative variance for each system.

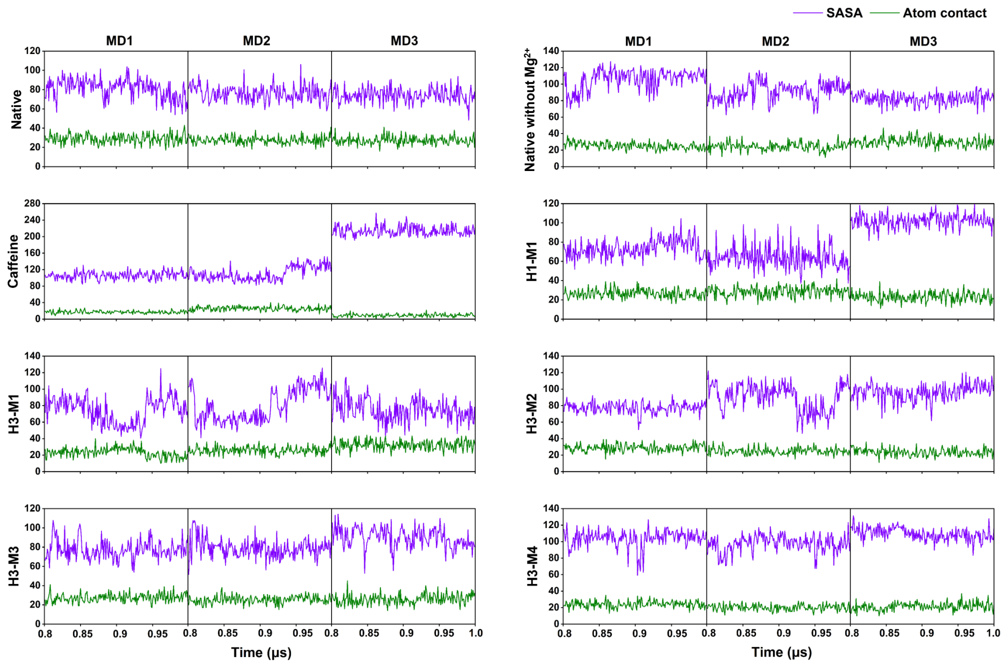

Figure S7: Ligand solvent-accessible surface area (SASA) and binding pocket atom contacts over 0.8 - 1.0 μs of simulation for all complexes. Each panel shows the time evolution of ligand SASA (purple) and the number of heavy atom contacts between the ligand and the binding pocket nucleotides (green) over three independent MD replicates. Contacts were defined using a heavy atom distance cutoff of 3.0 Å.
